# Hydrocarbon Frameworks with Long-Range Order Synthesized via Olefin Metathesis

**DOI:** 10.1021/jacs.5c22787

**Published:** 2026-02-19

**Authors:** Xuelin Sui, Chenxi Xiong, Jiaxing He, Sun Ho Park, Fan-cheng Kong, Zhuoliang Ying, Enhui Jiang, Philip C. Y. Chow, Yi Zhou, Keunhong Jeong, Osamu Terasaki, Yu Han, Nak Cheon Jeong, David Lee Phillips, Seungkyu Lee

**Affiliations:** † Department of Chemistry, 25809 The University of Hong Kong, Pokfulam Road, Hong Kong 99077, China; ‡ Department of Physics and Chemistry, 235496 DGIST, Daegu 42988, Republic of Korea; § Center for Basic Science, DGIST, Daegu 42988, Republic of Korea; ∥ Department of Mechanical Engineering, The University of Hong Kong, Pokfulam Road, Hong Kong 99077 China; ⊥ School of Physical Science and Technology, 387433 ShanghaiTech University, Shanghai 201210, China; # Department of Physics and Chemistry, 65767 Korea Military Academy, Seoul 01805, Republic of Korea; ∇ Electron Microscopy Center, 26467 South China University of Technology, Guangzhou 510640, China

## Abstract

Introducing long-range order into hydrocarbon covalent organic frameworks (COFs) remains a fundamental challenge because carbon–carbon bond-forming reactions are typically irreversible and lack effective error-correction mechanisms. Here, we demonstrate a molecular approach to enhance the crystallinity of a hydrocarbon framework. A fully hydrocarbon two-dimensional (2D) COF, HKU-50, was synthesized via reversible olefin metathesis. Polymerization of a vinylene-bearing monomer using the second-generation Grubbs catalyst initially yielded an amorphous network. Upon addition of *trans*-stilbene, secondary metathesis is activated, regenerating catalysts that are otherwise trapped within the growing polymer. This enables continuous bond exchange and error correction during framework formation. As a result, the system evolves toward its thermodynamically favored product, HKU-50, with long-range order. The resulting COF exhibits high thermal and chemical stability, along with enhanced photophysical properties, including red-shifted emission, prolonged fluorescence lifetime, and a 4-fold increase in photoluminescence quantum yield relative to its amorphous analogue due to its extended order. These findings demonstrate a molecular approach that actively controls the crystallinity of hydrocarbon frameworks without the use of supports or interfaces that induce partial ordering of the building units.

## Graphical abstract

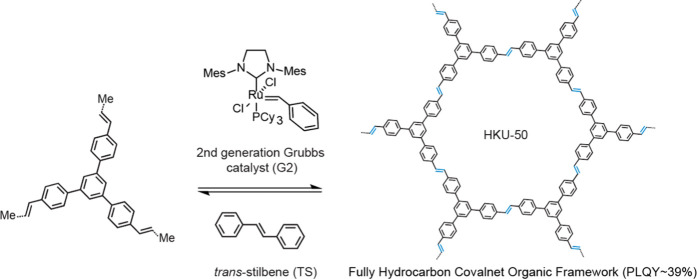

Achieving highly crystalline covalent organic frameworks (COFs) synthesized by carbon–carbon linkages remains a significant challenge.^1–9^ The carbon–carbon bond formation to synthesize COFs is generally irreversible near ambient conditions and is considered not suitable for crystallization.^10–15^ Such reactions lack an error correction process during crystallization and result in amorphous polymers.^10,12–16^

COFs with long-range orders have been successfully synthesized through carbon–carbon single or double bond formation reactions, including coupling reactions,^6,7,12,17^ aldol condensation,^18–25^ Horner–Wadsworth–Emmons reaction,^14,16^ cyanobenzene trimerization,^26^ and Knoevenagel reaction.^27–32^ In these reactions, heteroatoms such as N, F, and Cl—introduced to activate the starting materials or stabilize reaction intermediates—remain incorporated in the final framework backbone. Despite these significant advances, the synthesis of fully hydrocarbon composition frameworks and precise control over their crystallinity in homogeneous solution remain major challenges. COFs with carbon backbone can exhibit high chemical and thermal stability and unique physical properties that emerge from fully carbon-based orbital overlaps, including tunable band structures, enhanced charge delocalization, and anisotropic optical responses.^7–9,13,28,30–32^

Recently, the development of molecular approaches that control the kinetics of reversible bond formation during imine-based COF synthesis has substantially improved crystallinity, enabling precise structural determination and homogeneous synthesis of imine-linked COFs.^2,3,5^ Developing a similar molecular control strategies for COFs with fully carbon backbone could enable the synthesis of high-quality single crystals suitable for fundamental structural and property investigations, as well as scalable reactions compatible with industrial processes.

Here, we report a fully hydrocarbon two-dimensional (2D) COF, HKU-50 (HKU: The University of Hong Kong), whose crystallinity can be systematically tuned through olefin metathesis reactions. Using the second-generation Grubbs catalyst (G2), the vinylene-bearing monomer 1,3,5-tris­(4-propenylphenyl)­benzene (TPB-Me) polymerized to form HKU-50-amorphous (HKU-50-a). The relatively low crystallinity of the polymer was significantly increased by inducing secondary-metathesis reactions. Introducing a C = C bond-containing molecule, *trans*-stilbene (TS) as a secondary-metathesis-inducing reagent, markedly increases the crystallinity. Controlled experiments and in situ ^1^H and ^31^P NMR indicate that TS promotes secondary metathesis and releases framework-trapped catalysts back into solution, enabling continued exchange and error correction. The resulting COF exhibits high chemical stability under strongly acidic and basic conditions in both aqueous and organic media. Moreover, the COF displays intense photoluminescence under solid state, with photoluminescence quantum yields (PLQYs) of 38%, significantly higher than that (10%) of its amorphous analogues due to the extended order. These results establish a molecular approach to enhance the crystallinity of COFs with carbon backbone without the use of support or interfaces that partially orient building units to induce order.

Olefin metathesis is a reversible C=C bond exchange reaction catalyzed by transition-metal complexes such as Schrock and Grubbs catalysts (Figure 1a).^33–37^ Olefin metathesis has been extensively used in synthesizing polymers.^35,37–39^ However, it is well-known that during polymerization, the catalysts bind to the carbene terminal of polymers and precipitate together with the growing chains, resulting in possible deactivation.^40–42^ This behavior restricts the repeated bond-exchange events required for error correction for crystallization. We hypothesize that if the precipitated catalysts could be released back into solution, they would remain available for further metathesis cycles and thereby enable structural reorganization toward higher crystallinity.

**Figure 1:**
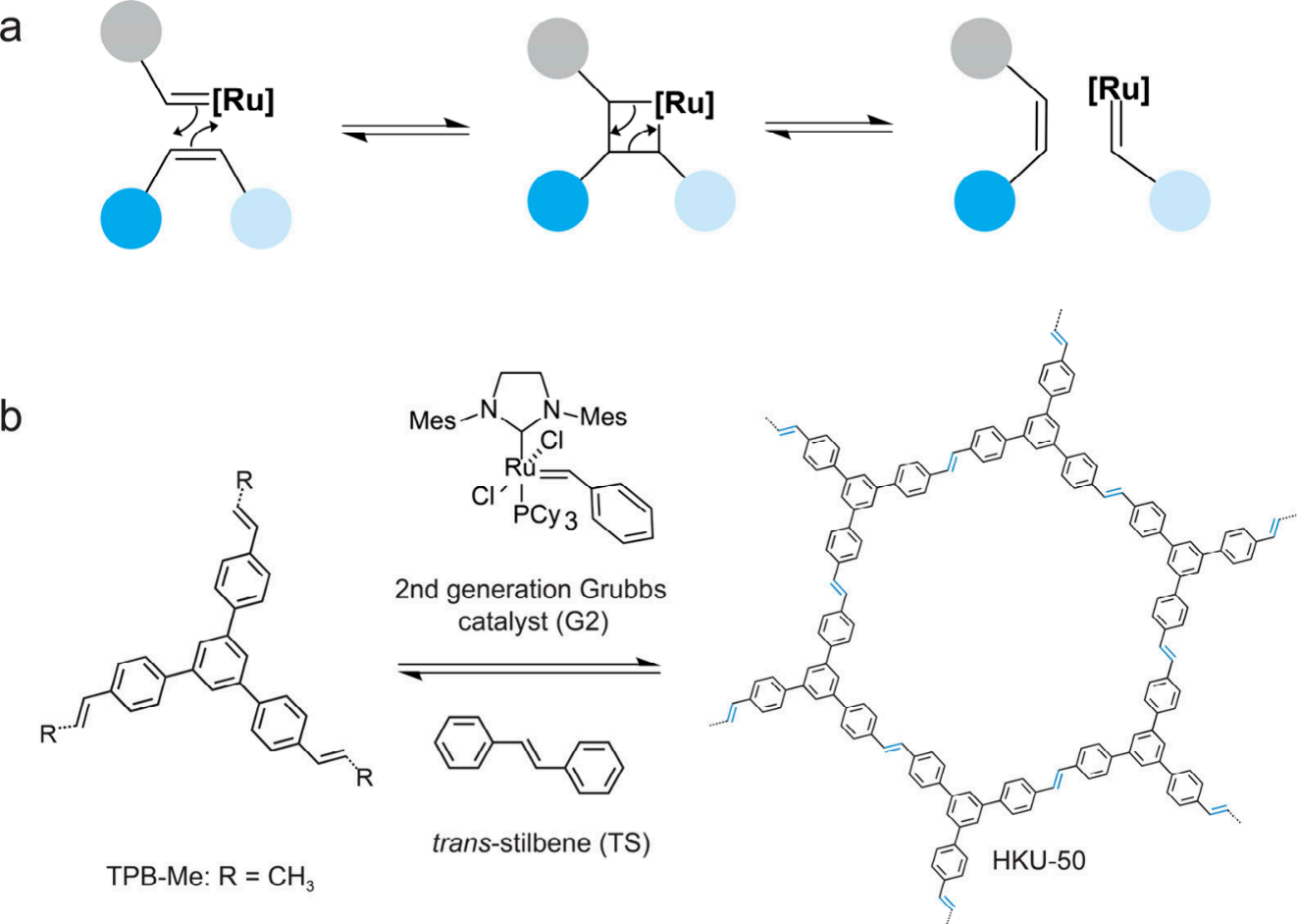
Synthesis of HKU-50 via olefin metathesis. (a) General mechanism of alkene metathesis mediated by a Grubbs catalyst. (b) Schematic representation of HKU-50 synthesis from the vinylene-bearing monomer, TPB-Me (R = Me), using the second-generation Grubbs catalyst and *trans*-stilbene.

To test this hypothesis, we assembled the trigonal monomer TPB-Me into the vinylene-linked framework, HKU-50, using G2 (Figure 1b). Under previously reported conditions for a structurally similar alkene building unit, metathesis produced only amorphous polymers, exhibiting a powder X-ray diffraction pattern (PXRD) with a broad peak at a low angle (Figure S9).^12^ We reasoned that introducing TS would promote secondary metathesis reactions between the C=C bond in TS and the carbene coordinated to the trapped catalyst (Figure 1b). Such exchanges would regenerate a soluble benzylidene-bearing catalyst, restoring its ability to mediate further bond exchange. In literature, secondary metathesis steps are known in small-molecule systems to shift equilibria toward more thermodynamically stable arrangements, for example, converting *cis*- to *trans*-alkenes, suggesting an analogous pathway for improving order in extended frameworks.^36,43–46^

Consistent with this mechanism, systematic optimization revealed that adding five equivalents of TS relative to G2 produced HKU-50 with sharp reflections in its PXRD pattern (Figure 2e and Figure S10), indicative of crystals with long-range order. Based on the reflection positions, a layered structure was modeled in a unit cell with a = 25.0548 Å and c = 3.8587 Å in the space group *P*3̅ (Figure 2a-c, and Table S1). The corrugated layers shown in Figure 2b were modeled based on the single crystal structure of TPB-Phenyl (TPB-Ph), synthesized via Horner-Wadsworth-Emmons reaction (Figure 2d).^14,47^ Large single crystals of TPB-Ph molecules were obtained and characterized by single-crystal X-ray diffraction (SXRD) (Figures S23 and S24 and Table S2). The refined structure displayed similar corrugated features arising from the torsional flexibility of the benzene rings and the rotation about the alkene linkages (Figure 2b and 2d).^48,49^ To further support the proposed structural model, we synthesized and fully characterized an imine-linked analogue, HKU-50-imine, which is expected to adopt a structure nearly identical to HKU-50 except for the presence of an imine nitrogen atom. The PXRD pattern of HKU-50-imine closely matches that of HKU-50 (Figures S27 and S28). In contrast, the PXRD patterns of TPB-Me, TPB-Ph, and HKU-50 differed from one another, confirming that HKU-50 is not a recrystallized form of either precursor species (Figure S10).

**Figure 2:**
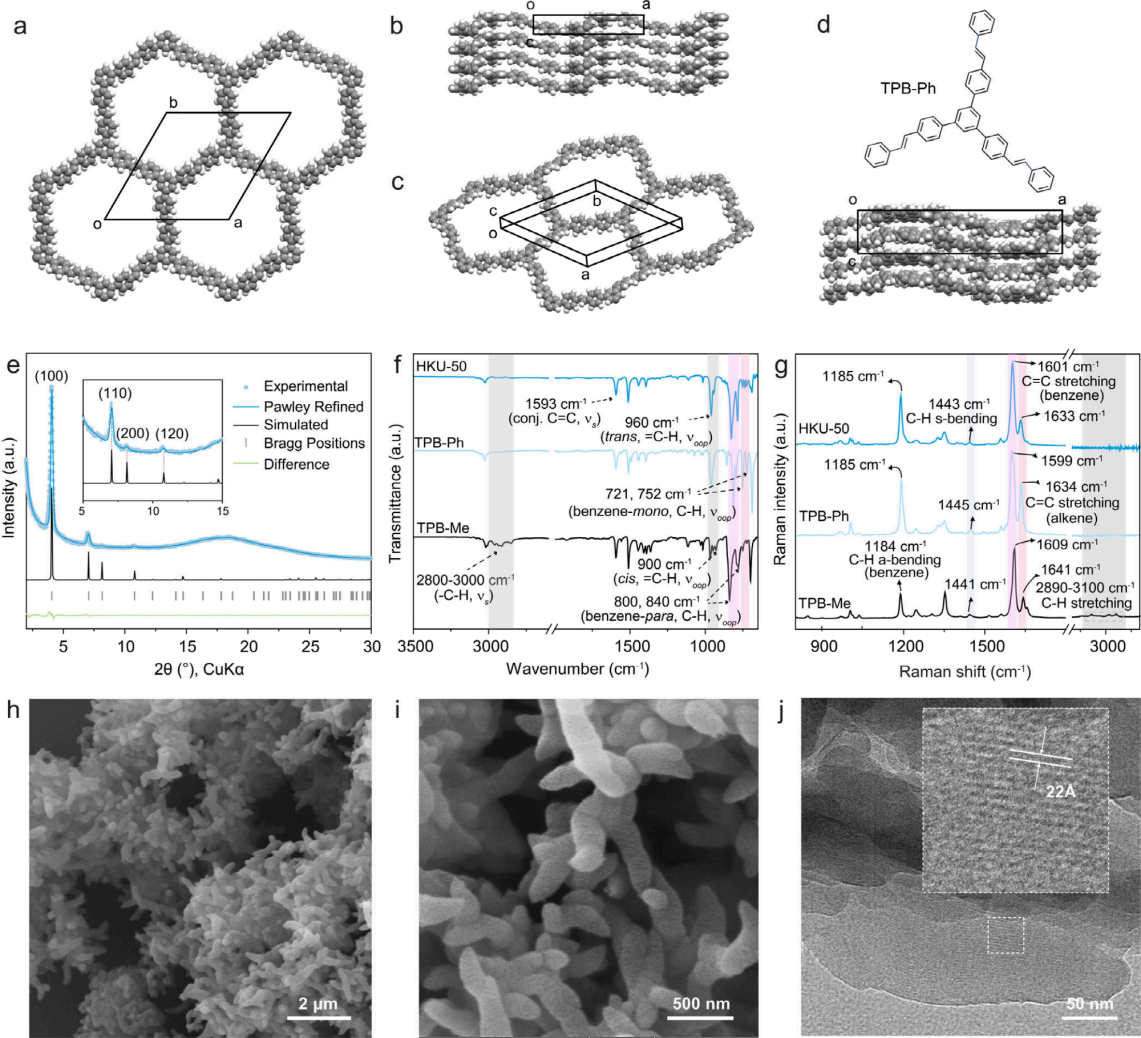
Characterizations of HKU-50. (a)–(c) Space-filling models of HKU-50 viewed along different orientations. (d) Space-filling model of TPB-Ph obtained from SXRD. (e) PXRD pattern of HKU-50. (f) FT-IR and (g) Raman spectra of HKU-50. (h) and (i) SEM images with different magnifications. (j) TEM image of HKU-50 showing lattice fringes corresponding to the (100) plane.

HKU-50 was characterized by Fourier-transform infrared (FT-IR) and Raman spectroscopy (Figure 2f and g). For comparison, the spectra of TPB-Me, the starting monomer, and TPB-Ph, a potential side product formed through metathesis between TPB-Me and TS, were shown in parallel. In the FT-IR spectra, the band at approximately 2900 cm^–1^, assigned to the terminal methyl C–H stretching vibration of TPB-Me, is almost absent in HKU-50, indicating the significant loss of the Me group during framework formation (Figure 2f). The two bands at 721 and 752 cm^–1^ in TPB-Ph corresponded to C–H out-of-plane vibrations of a monosubstituted benzene ring; as neither HKU-50 nor TPB-Me contains a monosubstituted benzene ring, these features are not shown in their spectra. In contrast, characteristic C–H out-of-plane vibrations of *para*-substituted benzene rings appeared prominently at approximately 800 and 840 cm^–1^ in both HKU-50 and TPB-Me. In the Raman spectra, the characteristic C–H stretching band near 2800–3100 cm^–1^ of the Me group of TPB-Me disappeared in the spectrum of HKU-50, again confirming the absence of the methyl group in HKU-50 (Figure 2g). The alkene C=C stretching vibration at 1641 cm^–1^ in TPB-Me shifts to a lower frequency, appearing near 1633 cm^–1^ in both TPB-Ph and HKU-50. This red shift is consistent with bulkier substitution around the C=C bond, as bulkier substituents lower the stretching frequency.

The morphology of HKU-50 was examined by scanning electron microscopy (SEM) (Figure 2h and 2i). The amorphous analogue, HKU-50-a, exhibits a spherical morphology typical of amorphous polymers (Figure S19). In contrast, HKU-50 displays an elongated, anisotropic morphology, suggesting a tendency to grow into hexagonal rods consistent with its internal symmetry. Transmission electron microscopy (TEM) analysis of the side facets of the elongated crystals reveals lattice fringes with a spacing of ∼22 Å, corresponding to the (100) plane and aligned along the long axis of the crystals (Figure 2j). This observation indicates that the anisotropic morphology originates from the crystal habit dictated by the framework symmetry. At a higher magnification, an interlayer spacing of ∼3.7 Å, assigned to the (001) plane, is also observed (Figure S20). These results suggest the possibility that, with further optimization, the crystallization conditions may yield single crystals with well-defined hexagonal-rod morphology.

The thermal stability of HKU-50 was evaluated by thermogravimetric analysis (TGA) under N_2_, revealing that the material remains stable up to approximately 400 °C (Figure S16). Its chemical stability was examined by immersing and stirring HKU-50 in common organic solvents (NMP, hexane, and DMF) as well as in strongly acidic and basic conditions in both aqueous and organic media for 2 weeks with stirring. The structural integrity of the treated samples was assessed by PXRD and FT-IR (Figures S21 and S22). Across all conditions tested, HKU-50 retains its crystallinity and chemical composition, as evidenced by the largely unchanged PXRD patterns and FT-IR spectra.^17–32^

We investigated the behavior of G2 in the presence of TS to examine how TS influences crystallinity. Two HKU-50 synthesis solutions were prepared under identical conditions, one containing TS and the other without TS. The supernatants of both solutions were analyzed by in situ ^1^H and ^31^P NMR (Figure 3a and Figure S33) to monitor G2 and Ru active species derived from it. In the reaction containing TS, a noticeable amount of G2 remained in solution even after 24 h, whereas in the reaction without TS, the concentration of G2 in the supernatant decreased sharply within 2 h (Figure 3a and Figure S33). These results indicate that TS prolongs the availability of G2 (or Ru active species) in solution. We hypothesize that TS slows the precipitation of G2 (or active Ru species) by competing with alkene units in solution and regenerating free G2 that has been trapped with the polymer. The prolonged presence of G2 (or active Ru species) might enable continued error-correction during framework formation. It is also possible that the competition between TS and alkene species might slow down the nucleation process and enhance crystallinity as reported in modulated imine COF synthesis.^2,3^

**Figure 3:**
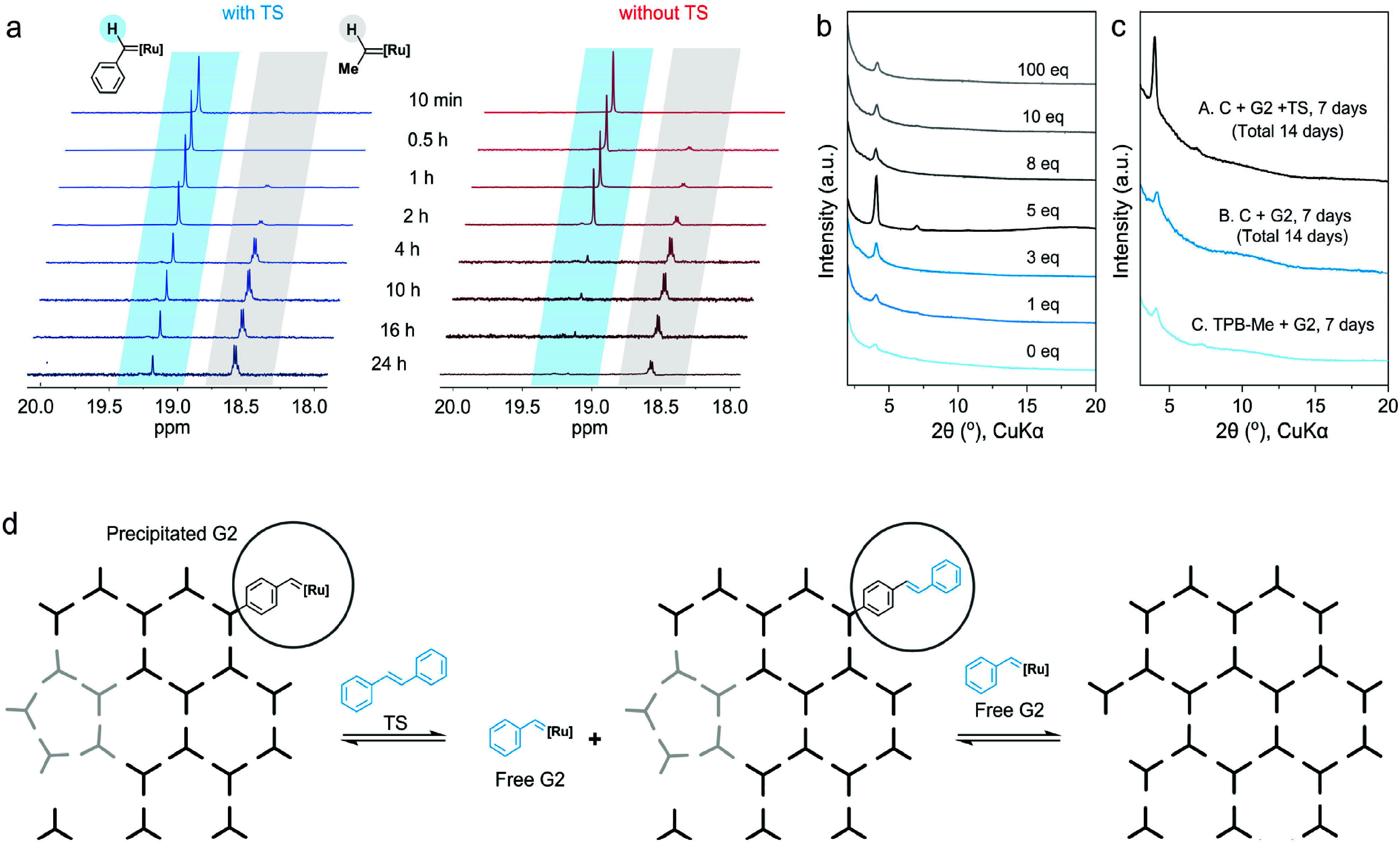
Role of *trans*-stilbene in promoting crystallinity of HKU-50. (a) In situ ^1^H NMR analysis of reaction supernatants with and without *trans*-stilbene (TS). (b) PXRD patterns of materials obtained using different TS loadings. (c) PXRD patterns from the sequential TS addition experiment. (d) Schematic illustration of the secondary metathesis–driven error-correction mechanism.

The crystallinity of the resulting materials was then evaluated by PXRD while varying the amount of TS from 0 to 100 equiv relative to G2. The crystallinity increases progressively as the TS amount approaches ∼5 equiv; however, further increasing TS to 100 equiv results in diminished crystallinity, indicating that TS enhances ordering only within an optimal range (Figure 3b). We attribute the improvement at low TS loadings to the release of precipitated G2, which facilitates secondary metathesis–driven error correction. At excessively high TS concentrations, TS may compete with TPB-Me, significantly slowing the polymerization rate and hindering effective linkage formation.

In the absence of TS, the reaction yields only the amorphous analogue HKU-50-a, even when the reaction time is extended to 7 days (Figure 3c). When G2 and TS were added to precipitated HKU-50-a and the mixture was heated at 60 °C for an additional 7 days, the resulting material displayed substantially enhanced crystallinity. This behavior suggests that ordering is thermodynamically driven, consistent with the reversible nature of olefin metathesis and the role of secondary metathesis in error correction. To further support this thermodynamic process, we examined the molecular metathesis behavior of *cis*-stilbene (CS) with G2 in the absence of TPB-Me, allowing direct observation of isomerization dynamics (Figure S32).^43–46^ The molecular experiment also support that secondary metathesis can correct misaligned or *cis*-configured linkages during crystallization. Taken together, these results support a crystallization mechanism in which TS slows and reverses G2 precipitation, thereby maintaining G2 and active Ru species in solution for an extended period. The sustained availability of the catalysts enables secondary metathesis–driven error correction, ultimately promoting the high crystallinity of HKU-50 (Figure 3d).

In general, long-range order in crystals can influence π-conjugation between linked building units.^48–52^ To investigate the extended conjugation in HKU-50, the optical properties of TPB-Me, TPB-Ph, HKU-50-a, and HKU-50 were examined by confocal microscopy, ultraviolet–visible diffuse reflectance spectroscopy (UV–vis DRS), steady-state fluorescence spectroscopy (SSFS), and femtosecond fluorescence up-conversion (fs-FU) spectroscopy. Confocal fluorescence imaging shows that HKU-50 exhibits stronger photoluminescence than amorphous HKU-50-a, suggesting enhanced conjugation in the crystalline material (Figure 4a).^51,52^ UV–vis DRS spectra display a clear red shift from TPB-Me (333 nm) and TPB-Ph (350 nm) to HKU-50-a (381 nm) and HKU-50 (396 nm) (Figure S29). Solid-state emission spectra also show progressive red shifts from TPB-Me (406 nm) to TPB-Ph (438 nm) and to HKU-50-a (439/464 nm) and HKU-50 (442/466 nm) (Figure 4b). Notably, relative to amorphous HKU-50-a, HKU-50 shows red-shifted emissions, consistent with more effective conjugation in HKU-50. These results collectively support that HKU-50 possesses the most extended conjugation among the tested materials.^51,52^

**Figure 4:**
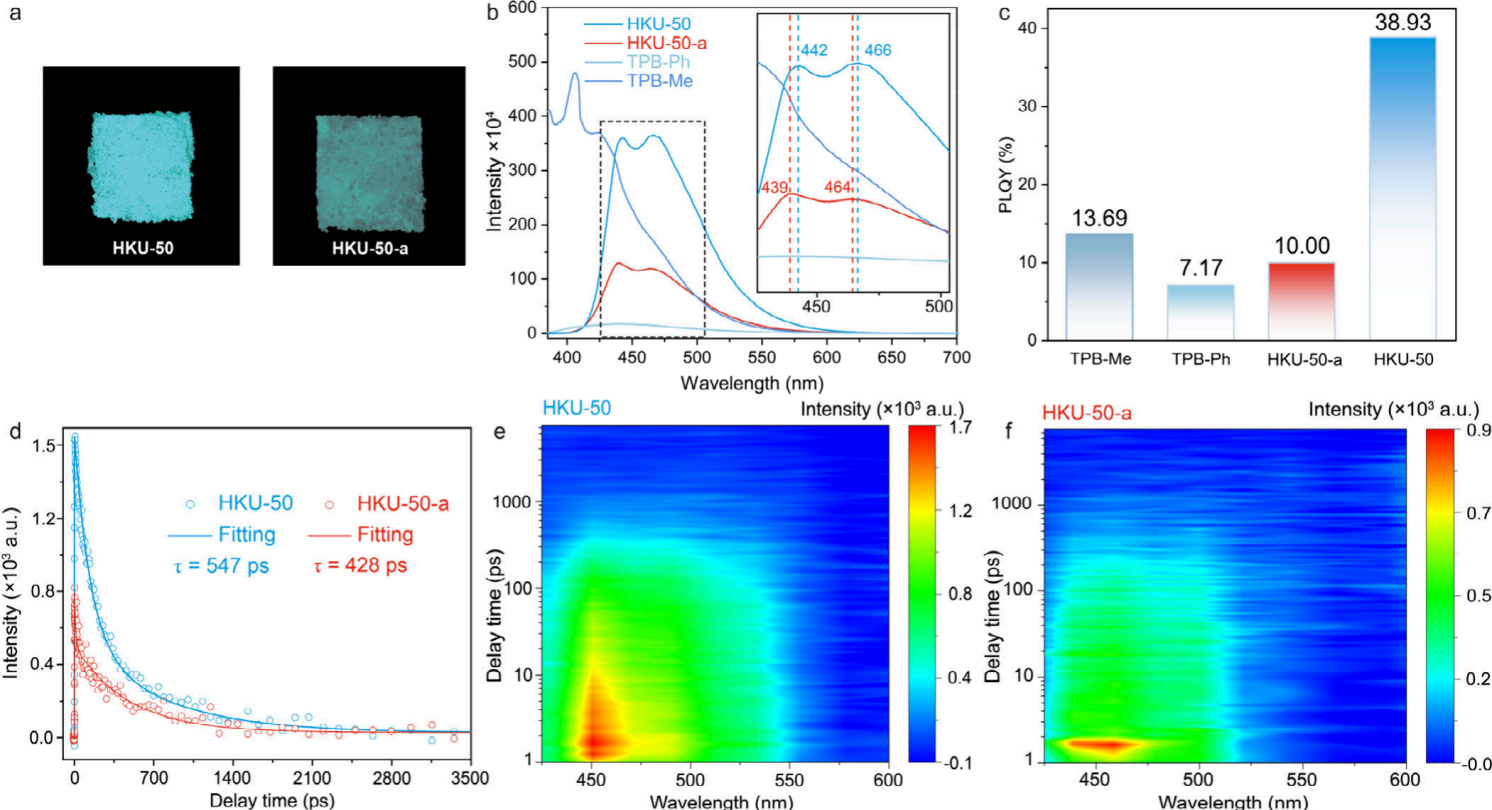
Photoluminescence comparison between amorphous HKU-50-a and crystalline HKU-50. (a) Confocal fluorescence microscope image of HKU-50 and HKU-50-a. (b) SSFS emission spectra of TPB-Me, TPB-Ph, HKU-50-a, and HKU-50 in solid-state. (c) Solid-state PLQYs of the four materials. (d) Fluorescence decay kinetics and biexponential fitting curves for HKU-50 and HKU-50-a. (e) and (f) fs-FU time–wavelength mapping of HKU-50 and HKU-50-a, respectively.

HKU-50 also shows a much higher solid-state photoluminescence quantum yield (PLQY) (38.9%) than HKU-50-a (10.0%) (Figure 4c). To further probe the origin of this enhancement, fs-FU measurements were conducted. The fluorescence decay profiles are well described by a biexponential model (Figure 4d), yielding an average lifetime τ of 547 ps for HKU-50, increased from 428 ps for HKU-50-a (Figure S37). fs-FU mapping further confirms the longer lifetime and broader transient emission of HKU-50 compared with HKU-50-a (Figure 4e and 4f). The extended fluorescence lifetime and higher PLQY of HKU-50 are attributed to its higher crystallinity and improved π-conjugation.^48–52^

These results establish a generalizable strategy for achieving long-range order in fully hydrocarbon frameworks through reversible carbon–carbon bond formation in homogeneous solutions. Based on established molecular alkene metathesis mechanisms, the incorporation of functional groups and alternative olefinic building units is, in principle, compatible with our COF synthesis methodology. However, monomers bearing bulky substituents or strongly electron-donating or electron-withdrawing groups may induce catalyst decomposition and reduced stereoselectivity during alkene formation.

## Supplementary Material



## Notes

Synthesis and characterization of the reported COF, including PXRD, experimental conditions, photoluminescence measurement, and synthesis and characterization of TPB-Ph and HKU-50-imine (PDF)
