# Supplementary material for: Hydrocarbon Frameworks with Long-Range Order Synthesized via Olefin Metathesis
Source: J Am Chem Soc. 2026 Feb 19;148(14):14693–9. doi: 10.1021/jacs.5c22787 (PMC13088230; doi:10.1021/jacs.5c22787)
Supplement: Supplementary file 1 [file ja5c22787_si_001.pdf]

# Supporting Information

## Hydrocarbon frameworks with long-range order synthesized via olefin metathesis

Xuelin Sui,[1,§] Chenxi Xiong,[1,§] Jiaxing He,[1,§] Sun Ho Park,[2,3] Fan-cheng Kong,[4] Zhuoliang Ying,[1] Enhui Jiang,[1] Philip C. Y. Chow,[4] Yi Zhou,[5] Keunhong Jeong,[6] Osamu Terasaki,[5] Yu Han,[7] Nak Cheon Jeong,[2,3,*] David Lee Phillips,[1,*] Seungkyu Lee[1,*]

[1]Department of Chemistry, The University of Hong Kong, Hong Kong SAR, China

[2]Department of Physics and Chemistry, DGIST, Daegu 42988, Republic of Korea

[3]Center for Basic Science, DGIST, Daegu 42988, Republic of Korea

[4]Department of Mechanical Engineering, The University of Hong Kong, Pokfulam, Hong Kong. China

[5]School of Physical Science and Technology, ShanghaiTech University, Shanghai 201210, China

[6]Department of Physics and Chemistry, Korea Military Academy, Seoul 01805, Republic of Korea

[7]Electron Microscopy Center, South China University of Technology, Guangzhou 510640, China

*Corresponding to nc@dgist.ac.kr, phillips@hku.hk, and skchem@hku.hk

## Table of contents


### Section S1. Materials and instrumentation

Section S1.1 Chemicals 3

Section S1.2 Instrumentation 3

### Section S2. Synthesis method

Section S2.1 Synthesis of TPB-Me and H 5

Section S2.2 Synthesis of TPB-Ph 9

Section S2.3 Synthesis of HKU-50 11

### Section S3. Characterization of HKU-50

Section S3.1 Powder X-ray diffraction 13

Section S3.2 $N_2$ isotherm measurements 18

Section S3.3 Thermogravimetric Analysis (TGA) 19

Section S3.4 Solid-state $^{13}C$ CP/MAS NMR spectroscopy 20

Section S3.5 SEM and TEM images of HKU-50 20

Section S3.6 Stability test of HKU-50 22

Section S3.7 Structure determination of TPB-Ph 24

Section S3.8 Synthesis and characterization of HKU-50-imine 27

Section S3.9 ICP-OES measurement of HKU-50 32

### Section S4. Crystallization study with stilbene

Section S4.1 Studies of the metathesis process in a molecular system 33

Section S4.2 PXRD study of the influence of the amount of TS on the crystallinity of HKU-50 36

### Section S5. Photoluminescence studies

Section S5.1 Photoluminescence measurement of HKU-50, HKU-50-a TPB-Me, and TPB-Ph 37

Section S5.2 Confocal fluorescence images of HKU-50 and HKU-50-a 39

### Section S6. Density functional theory (DFT) computational study

Section S6.1 Electronic structure analysis 40

Section S6.2 Vibrational analysis 40

Section S6.3. Coordinates of studied molecules 41

### Section S7. References

**Figure S1-S39, Table S1-S4**

## S1. Materials and instrumentation

### S1.1. Chemicals

1,3,5-tris(4-formylphenyl)benzene (purity ≥ 97 %), 1,3,5-tris(4-aminophenyl)benzene (purity ≥ 97 %), methyltriphenylphosphonium bromide (purity ≥ 99 %), ethyltriphenylphosphonium bromide (purity ≥ 98 %), potassium carbonate ($K_2CO_3$, 99 %), sodium hydride (NaH) (60% dispersion in mineral oil), diethyl benzylphosphonate (purity ≥ 98 %), Grubbs 2$^{nd}$ generation catalyst (G2, purity ≥ 95 %), trans-stilbene (purity ≥ 95 %), cis-stilbene (purity ≥ 95 %), dichlorobenzene (DCB, 99.9%, extra dry, with molecular sieves, water ≤ 30 ppm), dichloroethane (DCE, 99.9%, extra dry, with molecular sieves, water ≤ 30 ppm), mesitylene (Mes, purity ≥ 95 %), and N-methyl-2-pyrrolidone (NMP, purity ≥ 95 %) were purchased form Energy Chemical Co. Ltd. Sodium sulfate ($Na_2SO_4$, anhydrous, 99 %) was purchased from 3A Materials. Chloroform (99 %, GR), dichloromethane (DCM, 99%, GR), methanol (99 %, GR), hexane (95%, GR), and acetone (99.7%, HPLC) were purchased from Duksan Pure Chemicals Co. Ltd. Dimethyl sulfoxide (DMSO, 99%, AR) dioxane (99%, AR), toluene (Tol, 99%, AR), and tetrahydrofuran (THF, 99%, AR) were purchased from RCI Labscan Group Co. Ltd. All chemicals were used as received without further purification.

### S1.2. Instrumentation

A Tousimis Samdri PVT-3D critical point dryer was used to activate the samples. Liquid $CO_2$ was used to exchange the ethanol thoroughly by repeatedly filling and purging until some amount of dry ice emerged at the vent, while keeping the chamber temperature around 0 °C. After fully filling the chamber with liquid $CO_2$, it is heated to about 40 °C. After 30 mins, following slowly bleeding overnight, it can afford the desired samples. $N_2$ adsorption isotherms were recorded on Quantachrome quadrasorb volumetric gas adsorption analyzer. The powder X-ray diffraction (PXRD) analysis was performed on Rigaku MiniFlex600 X-Ray Diffractometer using Cu metal target radiation source at 40 kV and 15 mA. Nuclear Magnetic Resonance (NMR) spectra were recorded on Bruker Ascend 400, 500, and 600 NMR spectrometer at room temperature. Chemical shifts (δ) are reported in part per million (ppm) scale with respect to residual solvent signal as an internal reference. Multiplicities are reported as follows: s = singlet and d = doublet with corresponding coupling constants (J) in Hertz (Hz) and integration. $^{13}C$ cross polarization/magic angle spinning solid state nuclear magnetic resonance (CP/MAS ssNMR) experiments were performed on a Bruker AVANCE 400 WB spectrometer operating at 100.62 MHz for $^{13}C$ using a double resonance 4 mm MAS NMR probe and a sample spinning rate of ad. The samples were analyzed using a TESCAN MAIA3 XMH model high-

resolution Schottky FE-SEM. A beam intensity of 10 and an accelerating voltage of 10 kV were used to optimize the imaging. Ruthenium content was determined by inductively coupled plasma mass spectrometry (ICP-OES, Agilent 720ES, Agilent Technologies, USA). High-resolution mass spectra (HRMS) were recorded on EI-TOF. Transmission electron microscopy (TEM) samples were prepared by dispersing the material in mesitylene, followed by sonication for 3 h and multiple solvent exchanges with ethanol. A droplet of the resulting suspension was deposited onto a carbon-coated copper grid. TEM imaging was carried out using a Glacios Cryo-TEM operated at 200 kV. Images were acquired under low-dose conditions using a Falcon 4 camera (resolution:  pixels; pixel size: μm). Samples for transmission electron microscopy observation were dispersed in mesitylene. A droplet of the suspension was transferred onto a carbon-coated copper grid. Observation was performed on a Glacios Cryo-TEM at 200 kV. Images were recorded with a Ceta-D camera under low-dose conditions. Raman was measured using DXRTM2xi Raman Imaging Microscope (Thermo Scientific, US) with the laser wavelength at 780 nm. The objective was 10× objective, NA 0.25, Olympus - long working distance lens (UIS2 LMPlanFL N). The Xeuss 3.0 HR small-angle X-ray scattering system is used for the simultaneous measurement of the scattering intensity at small scattering angles (SAXS) using 2D "Hybrid Single Photon Counting" detectors. Special features are the detector positions within the vacuum tube, which can be adjusted in all spatial directions, as well as the automatically adjustable beam collimation between 50 μm and 2000 μm beam size.

## S2. Synthesis method

### S2.1. Synthesis of TPB-Me and H

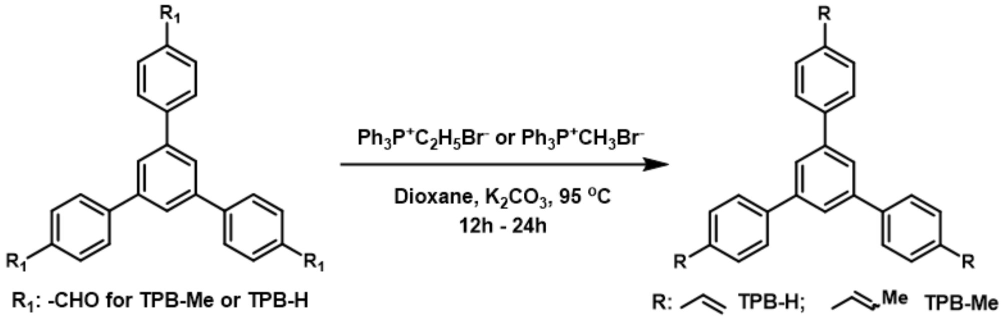


**Scheme S1.** General synthesis of TPB-H and TPB-Me.

**General procedure**: TPB-H and TPB-Me were synthesized based on the reported protocol with slight modifications.[1] A benzaldehyde derivative (1 mmol) was added to potassium carbonate (0.7 g, 5 mmol) and alkyltriphenylphosphonium bromide (3.3 mmol) in anhydrous 1,4-dioxane (20 mL), and heated at reflux for 12 to 24 hours. The reaction mixture was cooled, filtered and concentrated in vacuo. The residue was dissolved in the least amount of DCM, followed by purification via column chromatography (silica, 0% to 50% DCM in hexane) to give monomers (TPB-H, TPB-Me) as white or light yellow solid.

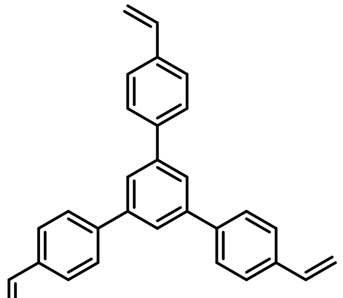

TPB-H

**TPB-H**: White solid (335mg, 87%). Prepared according to **General Procedure A** using 1,3,5-tris(4-formylphenyl)benzene and methyltriphenylphosphonium bromide with a reaction time of 12 hours.

**$^1$H NMR** (400 MHz, $CDCl_3$): δ 7.78 (s, 3H, Ar-*H*), 7.67 (d, J = 8.3 Hz, 6H, Ar-*H*), 7.53 (d, J = 8.3 Hz, 6H, Ar-*H*), 6.79 (dd, J = 17.6, 10.9 Hz, 3H, -C*H*=CH$_2$), 5.82 (d, J = 17.6 Hz, 3H, -CH=C*H*$_2$), 5.30 (d, J = 10.9 Hz, 3H, -CH=C*H*$_2$). **$^{13}$C NMR** (126 MHz, $CDCl_3$): δ 141.98, 140.48, 136.99, 136.43, 127.48, 126.79, 124.85, 114.15.

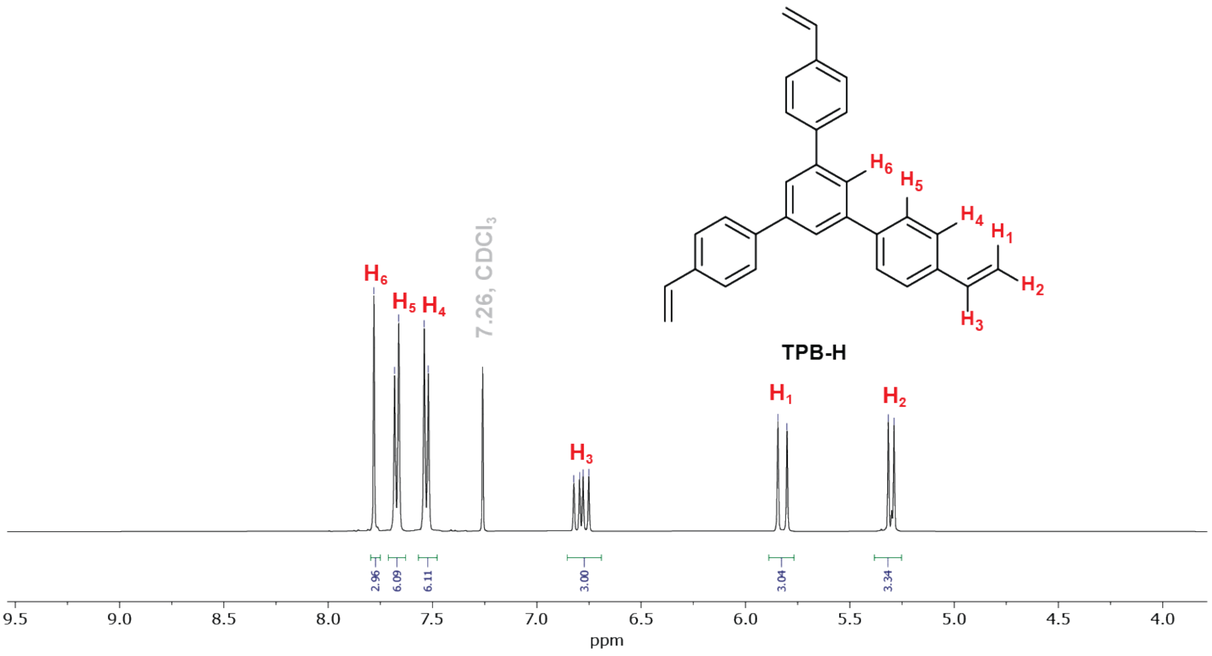


**Figure S1.** $^{1}H$ NMR Spectrum (400 MHz, $CDCl_3$, 298 K) of **TPB-H**.

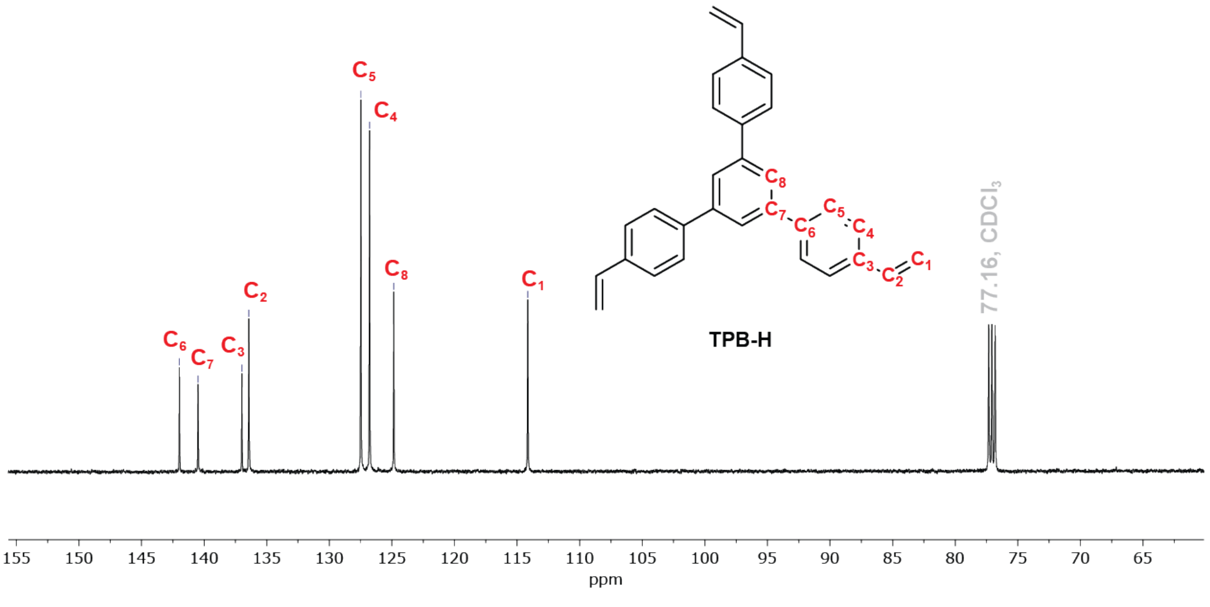


**Figure S2.** $^{13}C$ NMR Spectrum (126 MHz, $CDCl_3$, 298 K) of **TPB-H**.

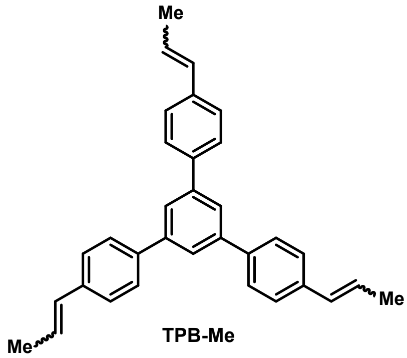


**Synthesis of TPB-Me**: TPB-Me was prepared according to **General Procedure**. 1,3,5-tris(4-formylphenyl)benzene (1 mmol) was added to potassium carbonate (0.7 g, 5 mmol) and ethyltriphenylphosphonium bromide (3.3 mmol) in anhydrous 1,4-dioxane (20 mL), and heated at reflux for 24 hours. The reaction mixture was cooled, filtered and concentrated in vacuo. The residue was dissolved in the least amount of DCM, followed by purification via column chromatography (silica, 0% to 50% DCM in hexane) to give **TPB-Me** (White solid, 380 mg, 89%).

**$^1$H NMR** (400 MHz, $CDCl_3$): δ 7.90 – 7.70 (m, 3H, Ar-*H*), 7.72 – 7.53 (m, 6H, Ar-*H*), 7.52 – 7.40 (m, 6H, Ar-*H*), 6.67 – 6.42 (m, 3H, -C*H*=CHCH$_3$), 6.40 – 6.18 (m, 1H, trans-CH=C*H*CH$_3$), 5.85 (dq, J = 11.7, 7.2 Hz, 2H, cis-CH=C*H*CH$_3$), 1.97 (dd, J = 7.2, 1.8 Hz, 6H, cis-CH=CHC*H*$_3$), 1.92 (dd, J = 6.6, 1.5 Hz, 3H, trans-CH=CHC*H*$_3$). **$^{13}$C NMR** (101 MHz, $CDCl_3$): δ 142.05, 142.03, 142.01, 139.46, 139.44, 139.16, 139.14, 137.34, 137.32, 137.01, 137.00, 130.60, 129.45, 129.38, 127.42, 127.23, 127.06, 126.34, 126.14, 126.12, 124.81, 124.75, 124.71, 124.65, 18.63, 14.85. **HRMS:** EI$^-$ Calculated: 426.2348, Measured: 426.2348.

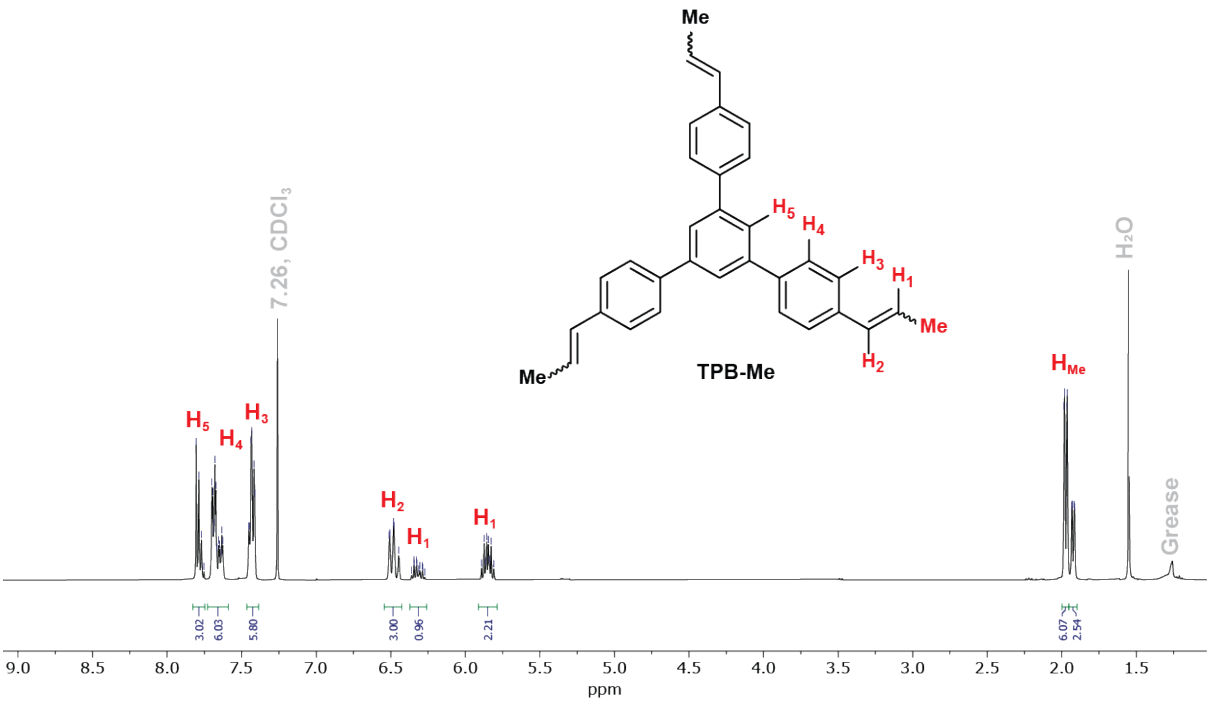


**Figure S3.** $^{1}H$ NMR Spectrum (400 MHz, $CDCl_3$, 298 K) of **TPB-Me**.

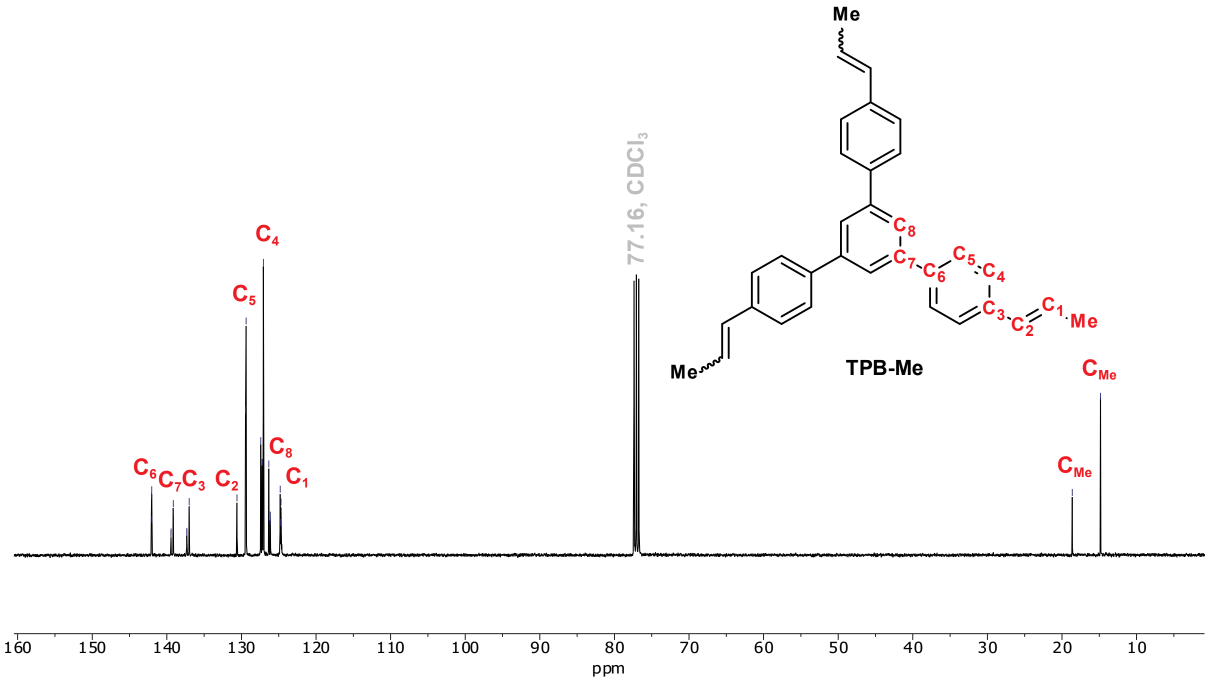


**Figure S4.** $^{13}C$ NMR Spectrum (101 MHz, $CDCl_3$, 298 K) of **TPB-Me**.

## S2.2. Synthesis of TPB-Ph

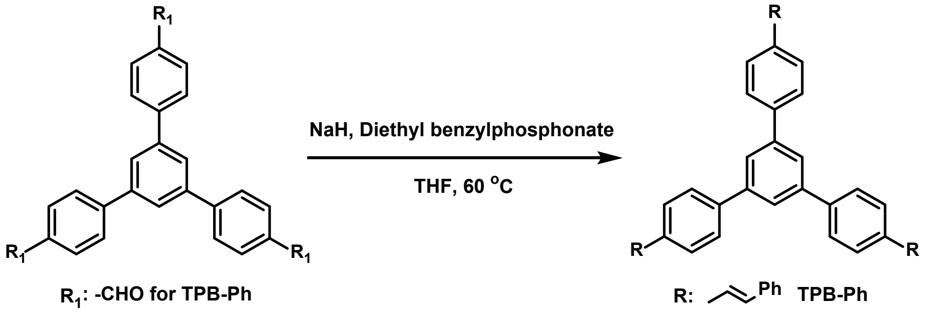


**Scheme S2.** General synthesis of Model Molecule (TPB-Ph).

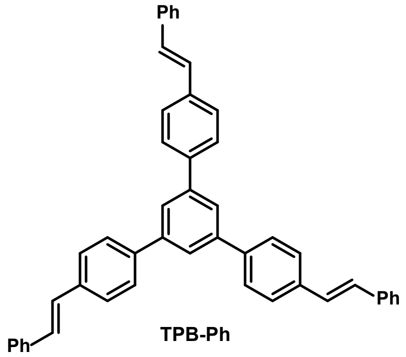


**TPB-Ph**: The Horner–Emmons reaction was modified to prepare **TPB-Ph** based on the reported protocol.[2] Sodium hydride (0.6 mmol) was added to phosphonate (0.4 mmol) in THF (3 mL) at room temperature. Different aromatic aldehydes (0.1 mmol) in THF (1 mL) were added to the mixture, which was further heated at 60 °C for 12 h. Then, after cooling, the crude mixture was slowly added to ice water, followed by filtration, and washing using 20 ml methanol to give a pale white solid (42 mg, 67%, TPB-Ph).

**$^1$H NMR** (400 MHz, $CDCl_3$): δ 7.86 (s, 3H, Ar-*H*), 7.76 (d, J = 7.4 Hz, 6H, Ar-*H*), 7.67 (d, J = 7.6 Hz, 6H, Ar-*H*), 7.59 (d, J = 7.7 Hz, 6H, Ar-*H*), 7.41 (t, J = 7.5 Hz, 6H, Ar-*H*), 7.32 (d, J = 7.4 Hz, 3H, Ar-*H*), 7.22 (s, 6H, -C*H*=CHPh). **$^{13}$C NMR** (126 MHz, $CDCl_3$): δ 141.95, 140.22, 137.31, 136.77, 128.98, 128.75, 128.16, 127.74, 127.61, 127.06, 126.59, 124.76. **HRMS:** EI$^-$ Calculated: 612.2817, Measured: 612.2827.

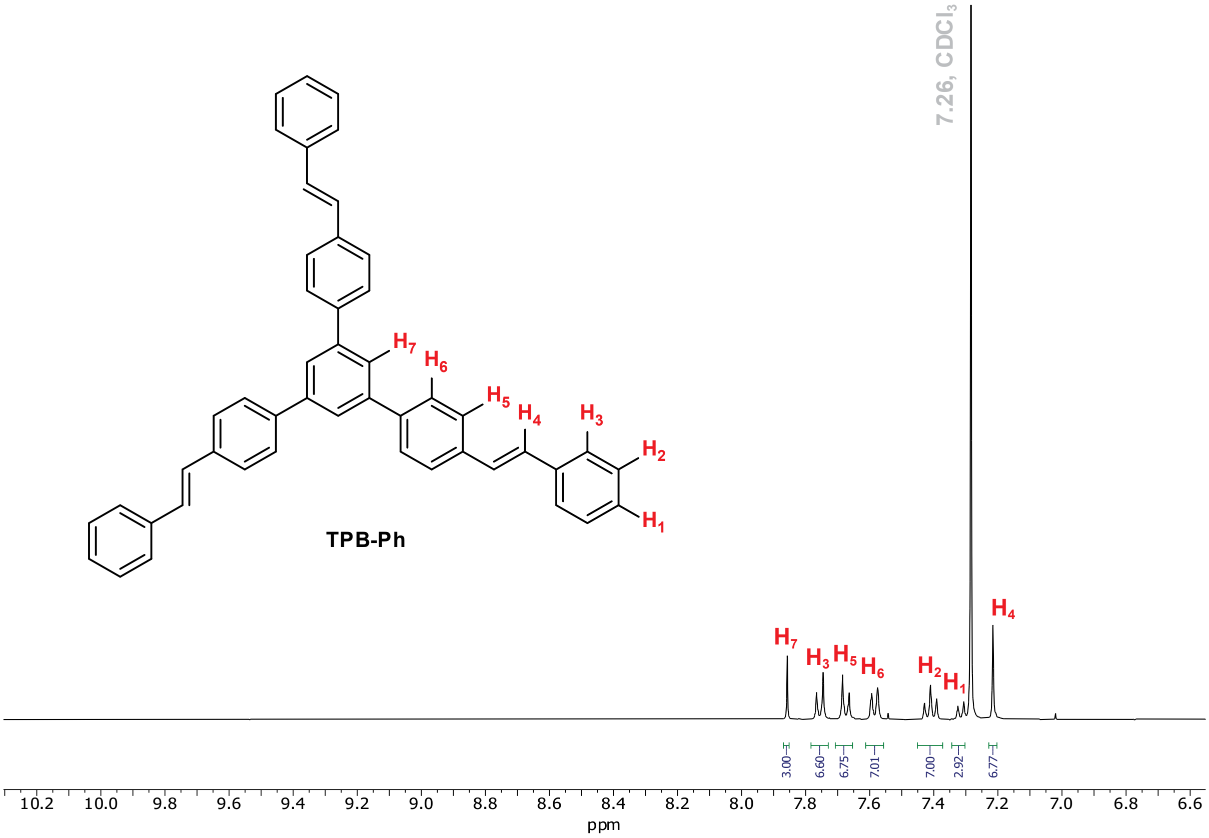

**Figure S5.** $^{1}$H NMR Spectrum (400 MHz, $CDCl_3$, 298 K) of **TPB-Ph**.

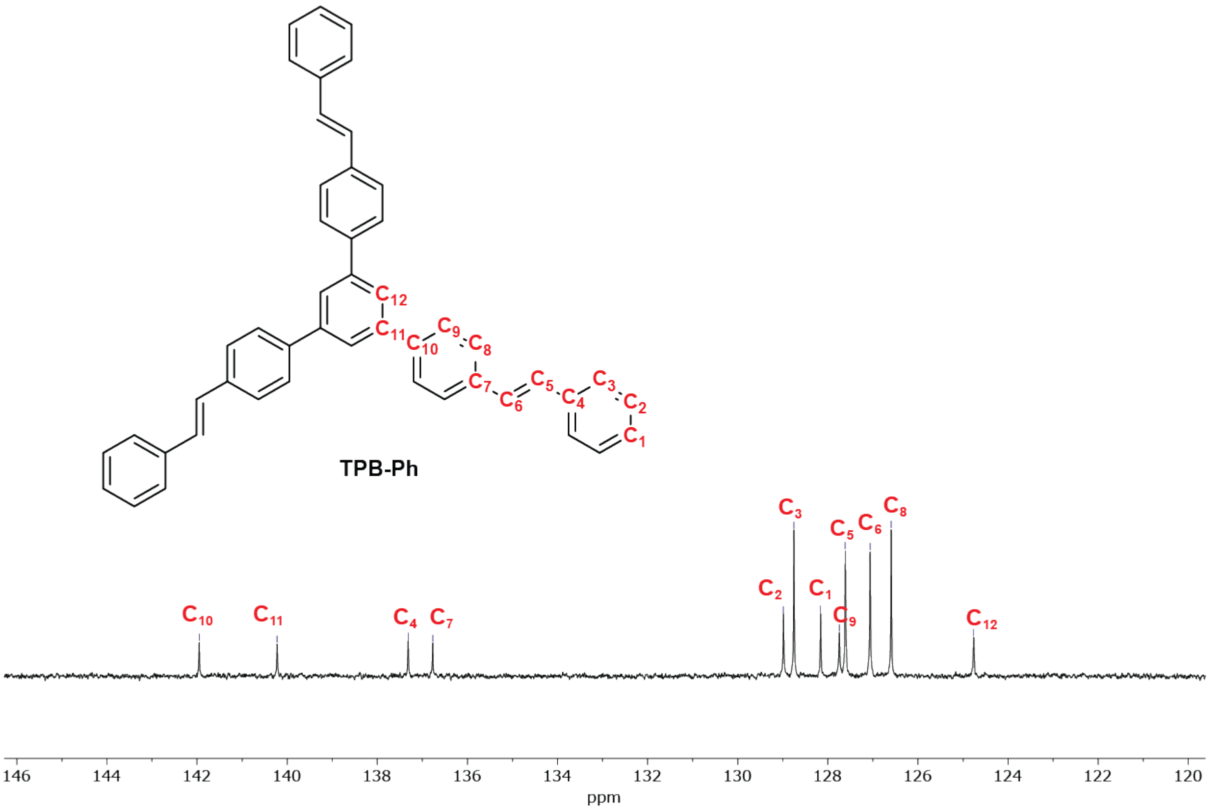

**Figure S6.** $^{13}C$ NMR Spectrum (126 MHz, $CDCl_3$, 298 K) of **TPB-Ph**.

**S2.3. Synthesis of HKU-50**

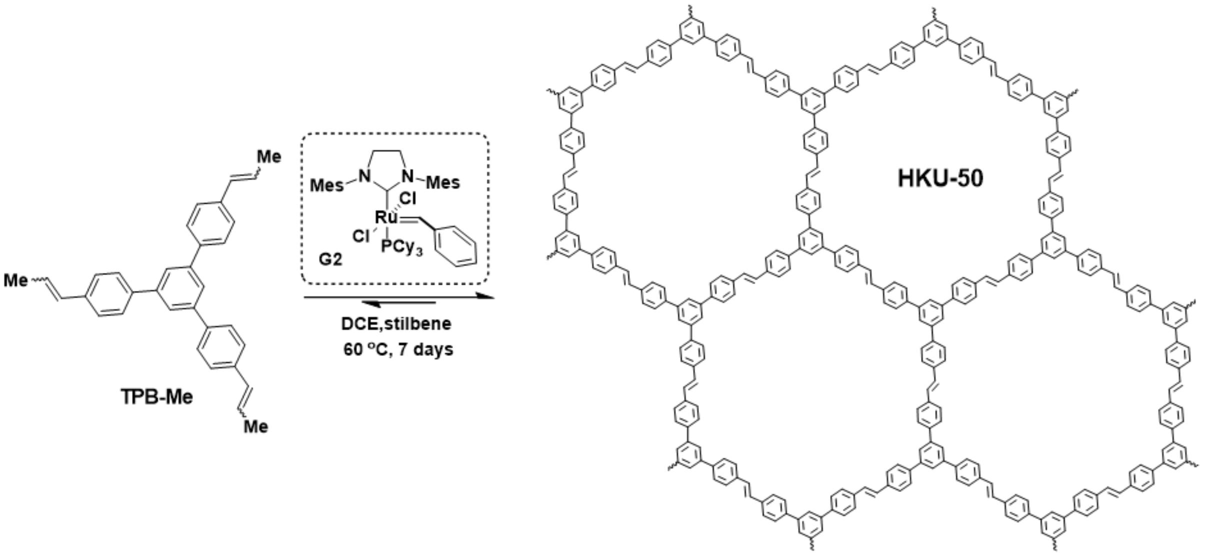


**Scheme S3.** Synthesis of HKU-50.

A borosilicate glass tube measuring "8 mm ×10 mm" ("i.d.× o.d.") was charged with TPB-Me (21.3 mg, 0.05 mmol), trans-stilbene (0.018 mmol, 3.2 mg), and G2 (4 μmol, 3.4 mg). DCE (0.5 ml) was added to the tube, followed by immediately degassing via freeze-pump-thaw three times before sealing the tube at internal pressure of 300 mtorr. Upon warming up to room temperature, the sealed tube was placed in a preheated oven at 60 °C and allowed to react undisturbedly for 7 days at desired temperature, when the light yellowish solid of HKU-20 formed in the tube. The tube was broken, and the solution was diluted with mixture of DMSO/THF (50 ml, v/v=1/10) and further washed with THF (10 ml, 3 times per day for 3 days). The THF-washed crystals were solvent-exchanged with anhydrous MeOH (10 ml, 3 times per day for 3 days). The MeOH-exchanged HKU-50 crystals were transferred into Kimwipe bag. Then, in a pre-cooled sample chamber, the anhydrous EtOH-exchanged sample was introduced and cooled to -10 °C. Liquid $CO_2$ was used to exchange the ethanol thoroughly by repeatedly filling and purging until some amount of dry ice emerged at the vent. To allow complete exchange, the chamber was filled with liquid $CO_2$ while keeping the chamber temperature around 0 °C. After the chamber was completely filled with liquid $CO_2$, the chamber was cooled to -10 °C and allowed to slowly warm up to 0 °C before purging liquid $CO_2$. The liquid level decreased, but it was still

high enough to cover the whole sample part. This process was repeated until the complete dry ice emerged at the vent. Then, the chamber was fully filled with liquid $CO_2$ before being heated to about 40 °C. During the heating, the pressure inside the chamber gradually increased to about 1400 psi to ensure the supercritical condition. The sample was allowed to stand in the supercritical $CO_2$ state for 30 min before slowly bleeding overnight to afford white crystals (11 mg, 51.6%).

**Synthesis of samples with low crystallinity (HKU-50-a)**

A borosilicate glass tube measuring "8 mm ×10 mm" ("i.d.× o.d.") was charged with TPB-Me (0.05 mmol), and G2 (4 μmol, 3.4 mg). DCB or DCE (0.5 ml) was added to the tube, followed by immediately degassing via freeze-pump-thaw three times before sealing the tube at internal pressure of 300 mtorr. Upon warming up to room temperature, the sealed tube was placed in a preheated oven at 60 °C and allowed to react undisturbedly for 7 days at desired temperature, when the light yellowish solid formed in the tube. The tube was broken, and the solution was diluted with a mixture of DMSO/THF (50 ml, v/v=1/10) and further washed with THF (10 ml, 3 times per day for 3 days). The THF-washed samples were solvent-exchanged with anhydrous MeOH (10 ml, 3 times per day for 3 days). The MeOH-exchanged samples were transferred into Kimwipe bag and activated via supercritical $CO_2$ as described above, yielding amorphous samples.

# S3. Characterization of HKU-50

## S3.1. Powder X-ray diffraction

The measurements were done using Rigaku MiniFlex600 X-Ray Diffractometer at room temperature using Cu metal target radiation source at 40 kV and 15 mA. The data were collected with the zero-background sample holder and scanned over the angular range from 2° to 30° (2θ) with a step size of 0.01. For common measurements, the crystals were prepared by soaking in methanol, mesitylene (Mes), and N-methyl-2-pyrrolidone (NMP), and mounted on the sample holder. However, through activation using direct vacuum drying or supercritical $CO_2$, the quality of PXRD is significantly reduced, with an obvious peak shift (Fig. S7). After putting the dried sample into the solvent for 2 hours, the quality of PXRD can be recovered (Fig. S8), as reported for flexible COF.[3]

The PXRD peaks at 4.07°, 7.04°, 8.14°, and 10.76° can be assigned to the (100), (110), (200), and (120) reflections, respectively (Figure S11). The experimental PXRD pattern matches the simulated profiles based on both AA and AB stacking models (Figures S12 and S13). Pawley refinement yielded satisfactory agreement factors for HKU-50 (Rp = 2.09% and Rwp = 3.16%). 001 reflection was not observed. The stacking may be disordered due to the corrugated layer structure predicted by our model, leading to a reduced π–π stacking reflection intensity. Besides the corrugated structures reported in our model to reduce the stacking intensity in our materials, our PXRDs were measured under wet conditions, which would make the 001 peak invisible, similar to the reported 2D imine COF measured in solvents.[4]

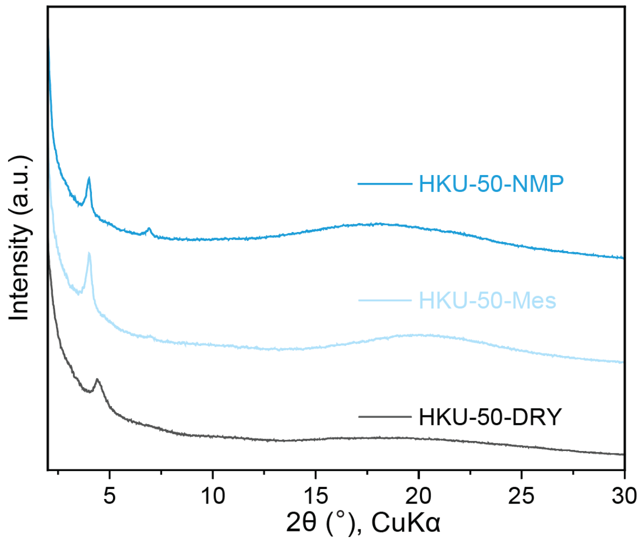


**Figure S7**. Experimental PXRD patterns of HKU-50 under dry, mesitylene suspension, and NMP suspension.

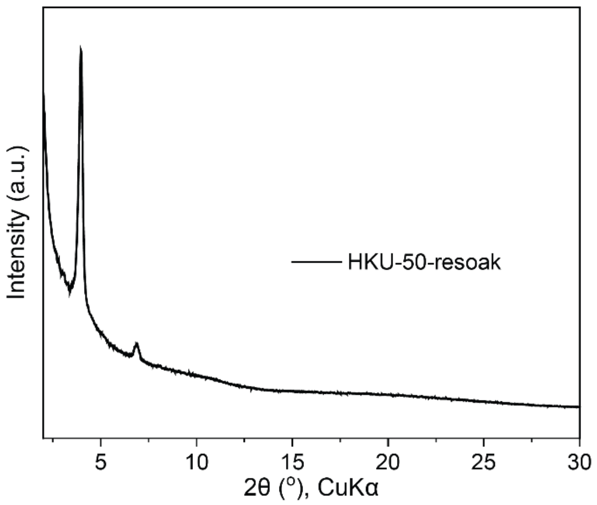


**Figure S8**. Experimental PXRD patterns of resoaked HKU-50. The dry samples were resoaked in methanol for 1 hour before PXRD measurement.

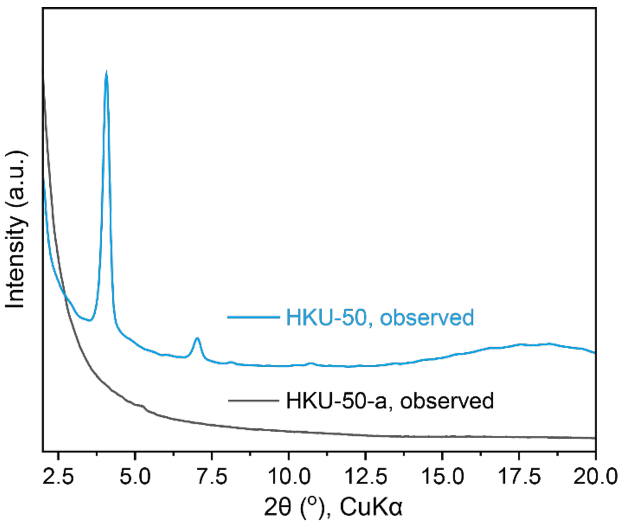


**Figure S9.** PXRD pattern comparison between HKU-50-a and HKU-50.

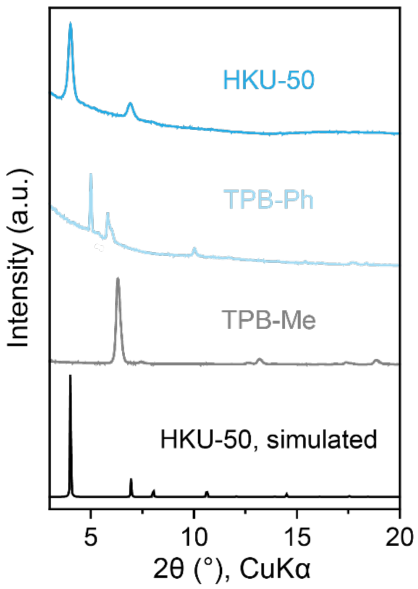


**Figure S10.** PXRD pattern comparison between of TPB-Me, TPB-Ph, and HKU-50.

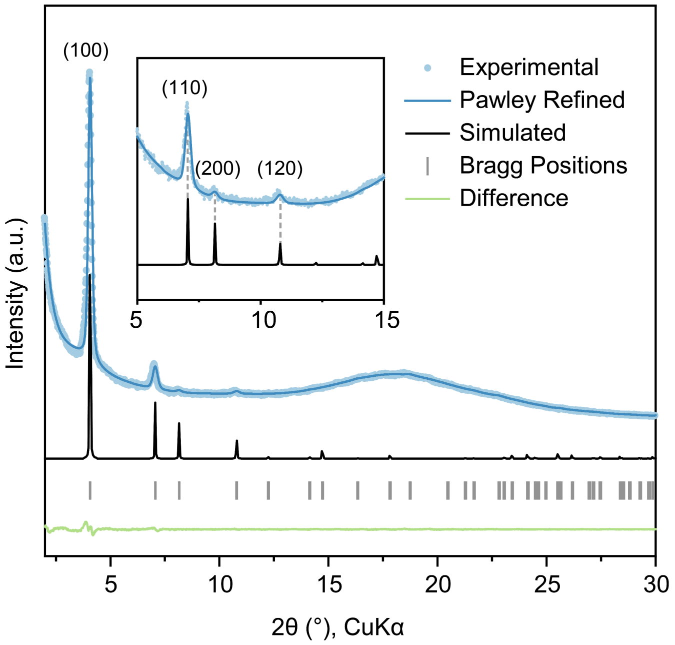


**Figure S11.** Experimental, Pawley refinement, and simulated powder XRD patterns of HKU-50 (Rp = 2.09% and Rwp = 3.16%).

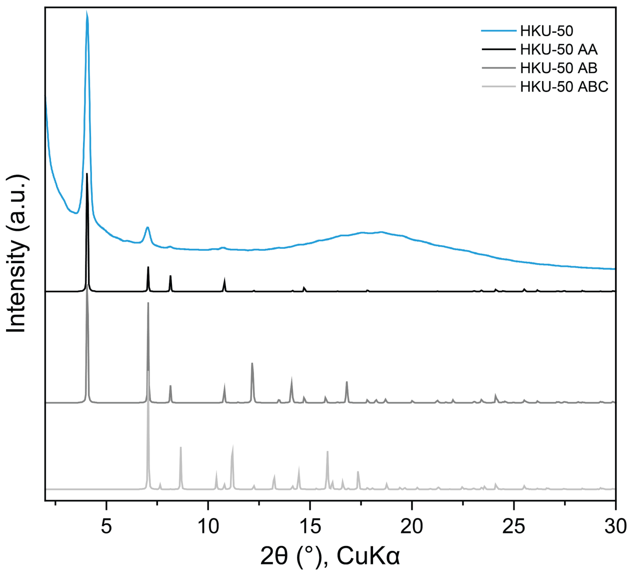


**Figure S12.** PXRD pattern analysis of HKU-50 under different stacking modes (AA, AB, and ABC).

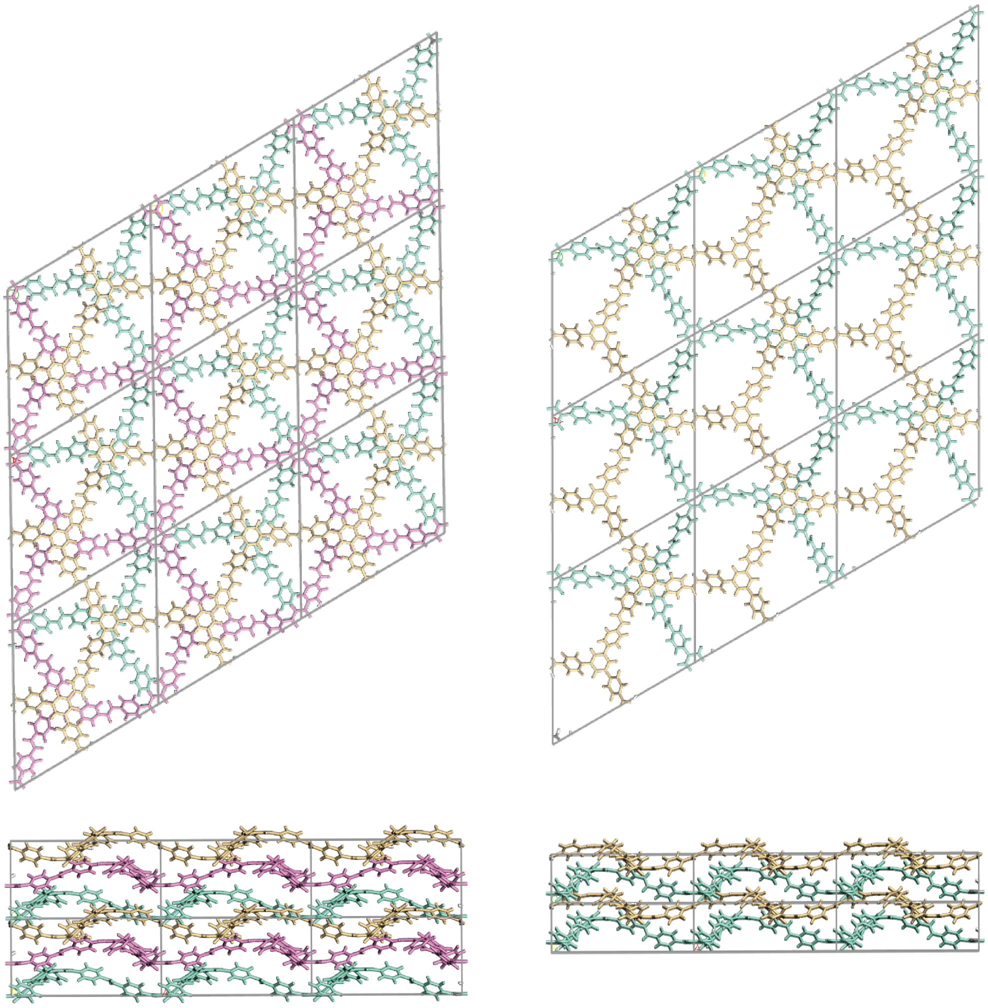

**Figure S13.** Simulated XRD patterns of HKU-50 with stacking mode as ABC (left) and AB (right).

**Table S1.** The fractional atomic coordinates and unit cell parameters of HKU-50, simulated based on PXRD of HKU-50.

| **HKU-50** | **Space group:** *P*-3 | | | | |
|---|---|---|---|---|---|
| | $a$ = 25.0548 Å, $c$ = 3.8587 Å | | | | |
| Atom site label | Atom site symbol | x | y | z | Atom site occupancy |
| C1 | C | 1.39752 | -1.30201 | -1.05489 | 1.00 |
| C2 | C | 1.36662 | -1.26835 | -1.04468 | 1.00 |
| C3 | C | 1.40123 | -1.20117 | -0.96635 | 1.00 |
| C4 | C | 1.46372 | -1.16452 | -1.05684 | 1.00 |
| C5 | C | 1.49793 | -1.10397 | -0.93949 | 1.00 |
| C6 | C | 1.47025 | -1.07772 | -0.73941 | 1.00 |
| C7 | C | 1.40691 | -1.11295 | -0.66721 | 1.00 |
| C8 | C | 1.37335 | -1.17407 | -0.77243 | 1.00 |
| C9 | C | 1.50951 | -1.01409 | -0.61115 | 1.00 |
| H10 | H | 1.48666 | -1.18222 | -1.21804 | 1.00 |
| H11 | H | 1.54635 | -1.07745 | -1.00645 | 1.00 |
| H12 | H | 1.38264 | -1.09511 | -0.52041 | 1.00 |
| H13 | H | 1.32618 | -1.20033 | -0.68453 | 1.00 |
| H14 | H | 1.44715 | -1.27794 | -1.04765 | 1.00 |
| H15 | H | 1.55605 | -0.99026 | -0.70874 | 1.00 |

## S3.2. $N_2$ isotherm measurements

The surface area and porosity of HKU-50 were determined using $N_2$ adsorption isotherms at 77 K on Quantachrome quadrasorb volumetric gas adsorption analyzer (Quadrasorb EVO). Before the experiments, around 0.1 g of adsorbents were pre-degassed overnight at 373 K using the Quantachrome XeriPrep™ degasser. Subsequently, a further in-situ degassing at 363 K for 1 hour was performed to ensure the complete removal of water and other impurities. The removal of impurities in COF samples was evaluated via $^1$H NMR tests of suspension via soaking the sample in $CDCl_3$ at 50 °C for 24 hours. Nitrogen ($N_2$) adsorption measurements reveal a relatively low surface area of 69 $m^2\ g^{-1}$ (Figure S14), which might be attributed to flexible structures, collapsed pores during activation, staggered configurations of the layers, and the presence of AB stacking.

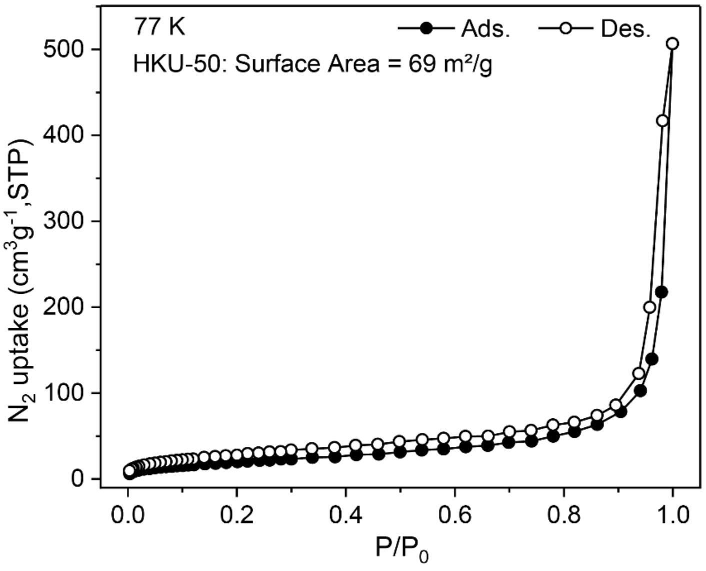


**Figure S14.** $N_2$ isotherm of HKU-50 measured at 77 K, surface area: 69 $m^2/g$.

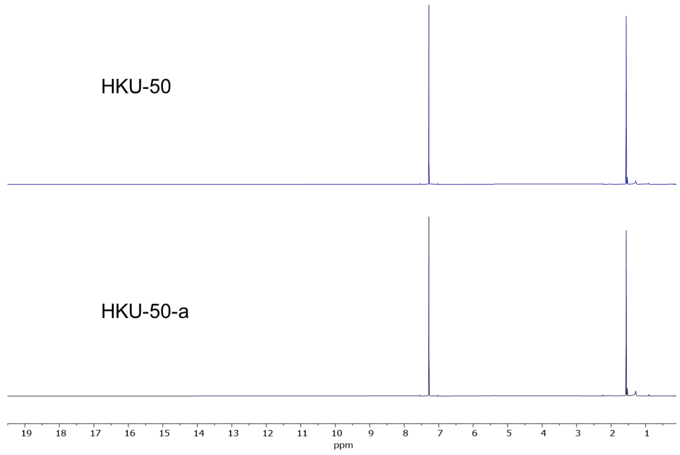


**Figure S15.** $^1$H NMR Spectrum of $CDCl_3$ suspension of purified HKU-50 and HKU-50-a.

## S3.3. Thermogravimetric Analysis (TGA)

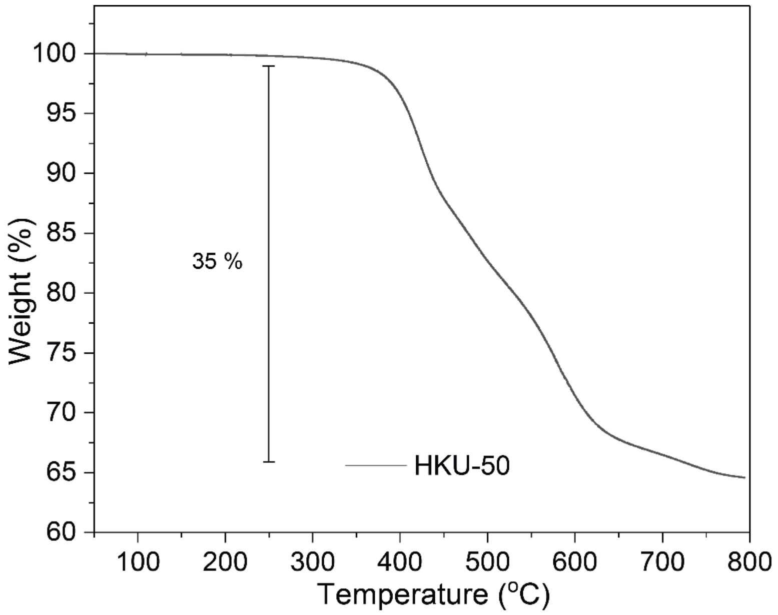


**Figure S16.** TGA data of HKU-50.

**Thermal stability test:** Activated HKU-50 powder (10.0 mg) was heated under Argon atmosphere at 200 °C and 350 °C for 1 hour. After cooling down, the COF was soaked in desired amounts of methanol, followed by characterization using PXRD under wet conditions.

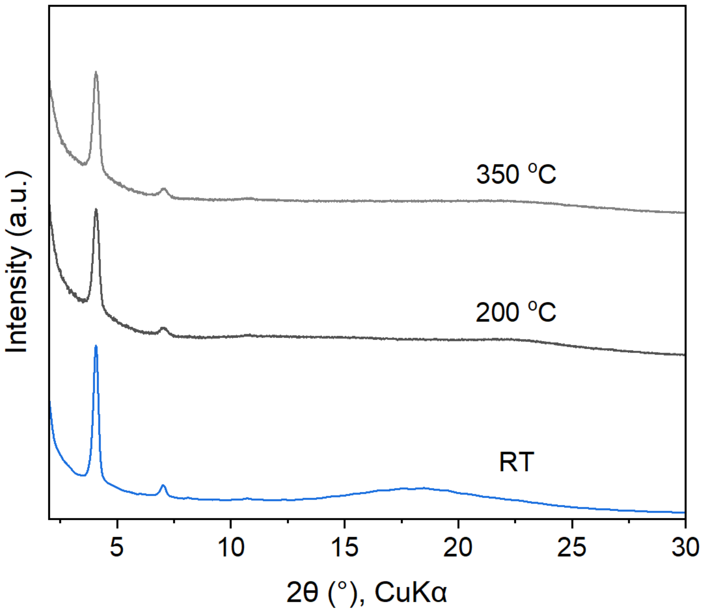


**Figure S17.** PXRD of HKU-50 after high temperature treatment at 200 °C and 350 °C.

### S3.4. Solid-state $^{13}C$ CP/MAS NMR spectroscopy

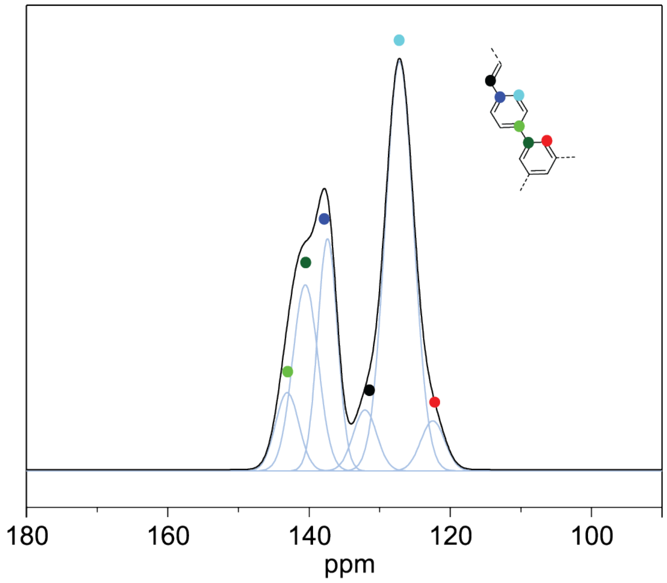


**Figure S18.** Solid-state $^{13}C$ CP/MAS NMR spectrum of HKU-50.

### S3.5. SEM and TEM images of HKU-50

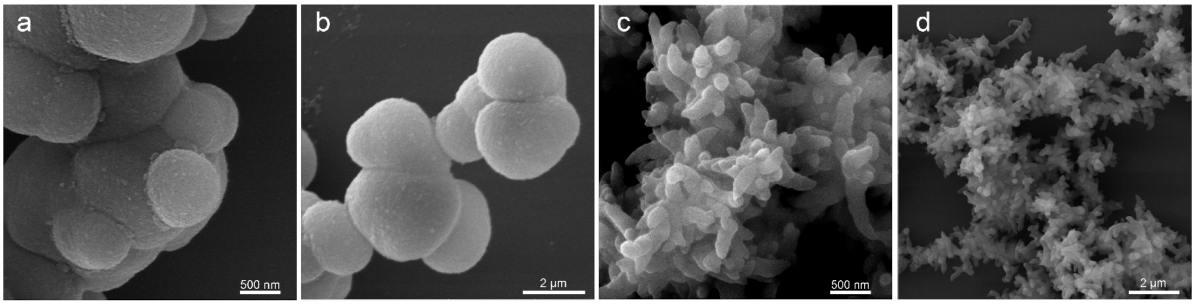


**Figure S19.** SEM image comparison between HKU-50-a and HKU-50. (a) and (b) SEM images of HKU-50-a with different magnifications. (c) and (d) SEM images of HKU-50 with different magnifications.

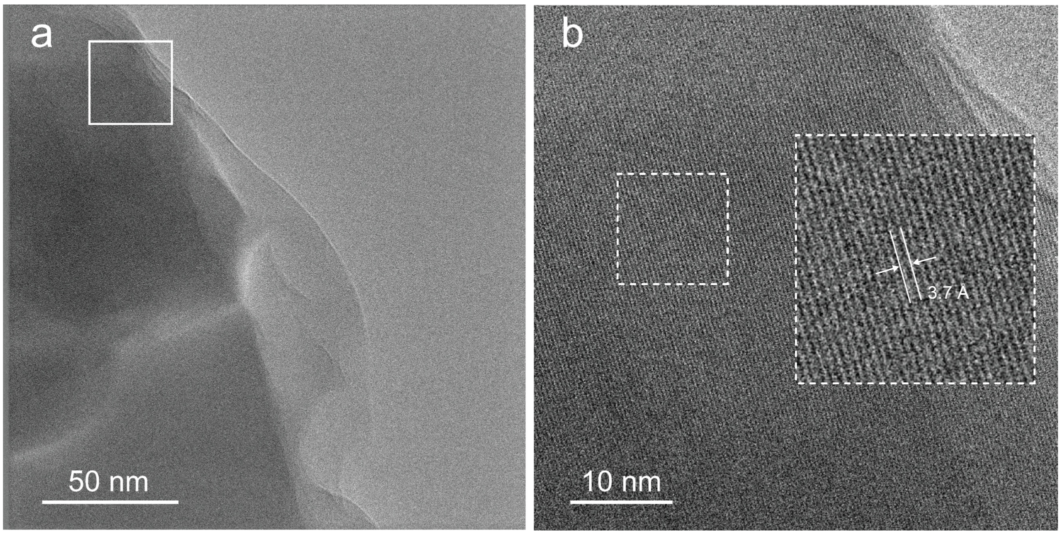


**Figure S20.** TEM images of HKU-50. (a) TEM image of HKU-50. (b) The magnified part of the selected region in (a) TEM image.

.

**S3.6. Stability test of HKU-50**

The chemical stability of HKU-50 was examined through two-week treatment (soaking the samples into liquid) using strong acids and bases, such as sodium hydroxide (6M NaOH, water), potassium hydroxide (saturated KOH, MeOH), hydrochloric acid (6M HCl, water), trifluoroacetic acid (6M in DMF, THF or $CH_3CN$), and an organic solvent such as NMP, hexane, respectively. The mixture was first diluted in water or methanol, filtered, neutralized with aqueous $NH_4OH$ or $NH_4Cl$ solution, washed with excess methanol, and reactivated prior to characterization by FT-IR and PXRD spectra. The spectra after treatment indicates that the crystallinity and skeletal structure of COFs are well maintained.

Activated HKU-50 powder (10.0 mg) was suspended in anhydrous tetrahydrofuran (THF, 5.0 mL) under argon and kept at -78 °C. A commercially available methyl lithium solution (1.6 mol/L in hexane, 0.1 mL) was added slowly over 5 minutes, stirred vigorously, and kept at −78 °C for 0.5 hours. The resulting mixture was diluted with THF, quenched with water, and neutralized with 1 mol/L aqueous $NH_4Cl$ solution. The COF was washed with excessive amounts of methanol, followed by characterization using PXRD and FT-IR.

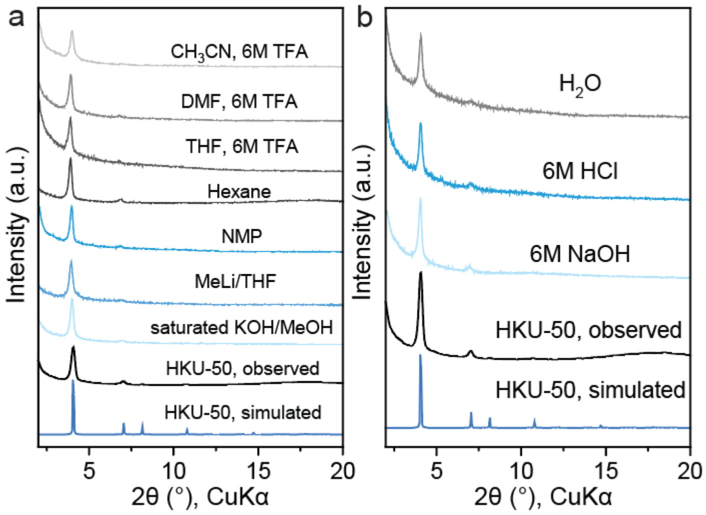


**Figure S21.** PXRD patterns of HKU-50 after various chemical stability tests. (a) PXRD of HKU-50 after treatment under various organic solvents and (b) under aqueous conditions.

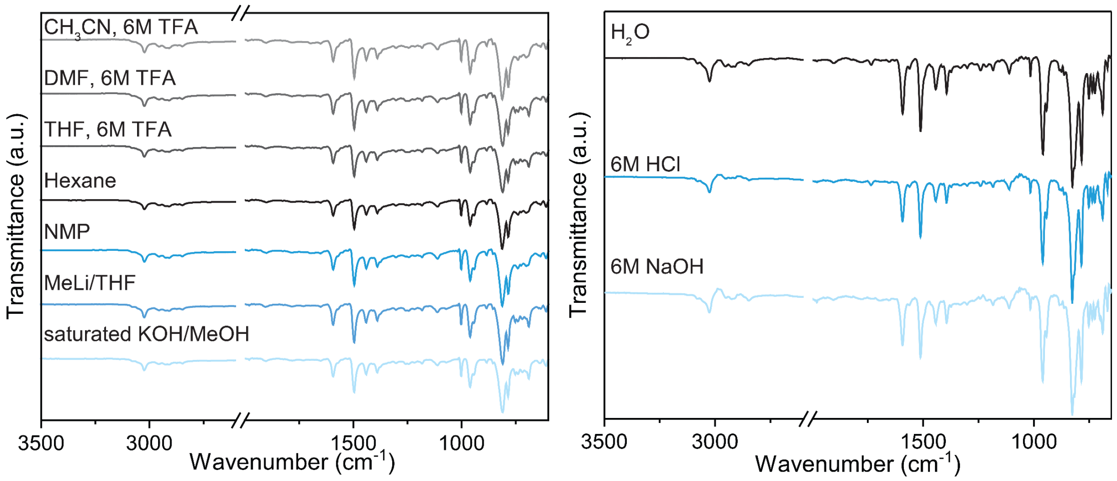


**Figure S22.** IR spectra of HKU-50 after treatment with various organic solvents and aqueous conditions.

**S3.7. Structure determination of TPB-Ph**

The SXRD data for TPB-Ph was collected using a copper X-ray source in Bruker D8 VENTURE. A cryogenic temperature of 100 K was used. A crystal (500 $\mu$m × 30 $\mu$m × 30 $\mu$m) of was coated with mineral oil and mounted on a 0.050 mm diameter loop. A data with a resolution of 1 Å was obtained. The SXRD data were processed with APEX6 software. Space group determinations and .ins file generations were done using XPREP software with .hkl files generated from SADABS, and inspecting reconstruction images of the data frames. Initial models for refinements were generated by direct method implemented in SHELXS and refined with SHELXL,[5] which is implemented in Olex2.[6] Non-hydrogen atomic positions were assigned first. Hydrogen atoms were attached to the model by riding models. The framework models were used for PLATON SQUEEZE[3]. The SQUEEZEd models were further refined with SHELXL until the parameters were converged. The structure was refined anisotropically. Crystal data and details of the structure refinement for single-crystal TPB-Ph are given in Table S3 and in the attached CIF file.

**Table S2.** Single crystal data and structure refinement parameters for TPB-Ph.

| **Name** | **TPB-Ph** |
|---|---|
| Empirical formula | $C_{50}H_{42}O$ |
| Formula weight | 658.83 |
| Temperature | 100 K |
| Crystal system | Orthorhombic |
| Space group | *Pbca* |
| a<br>b<br>c | 29.779(2) Å<br>7.5161(6) Å<br>35.164(3) Å |
| $\alpha$ | 90° |
| $\beta$ | 90° |
| $\gamma$ | 90° |
| Volume | 7870.5(11) Å$^3$ |
| Z | 8 |
| Density (calculated) | 1.112 g/cm$^3$ |
| Absorption coefficient | 0.491 mm$^{-1}$ |
| F(000) | 2800.0 |
| Crystal size | 0.5 × 0.03 × 0.03 mm$^3$ |
| Radiation | CuK$\alpha$ ($\lambda$= 1.54178 Å) |
| 2$\theta$ range for data collection | 5.026 to 46.10° |
| Index ranges | -32≤ h≤33, -7≤k≤7, -39≤ l≤39, |

| | |
|---|---|
| Reflections collected | 39997 |
| Independent reflections | 5886 [$R_{int}$ = 0.1125, $R_{sigma}$ = 0.0569] |
| Refinement method | Full-matrix least-squares on $F^2$ |
| Data/ restraints/ parameters | 5886/2/463 |
| Goodness-of-fit on $F^2$ | 1.134 |
| Final R indexes [I≥2σ(I)] | $R_1$ = 0.1389, $wR_2$ = 0.2539 |
| Final R indexes [all data] | $R_1$ = 0.2074, $wR_2$ = 0.2919 |
| Largest diff. peak and hole | 0.38/-0.27 e Å$^{-3}$ |

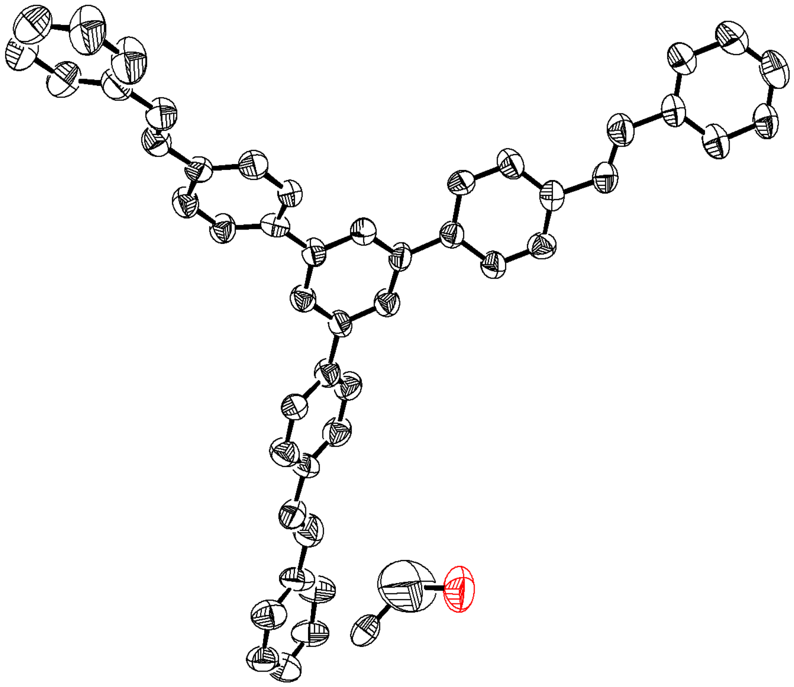

**Figure S23.** ORTEP drawing (50% probability) of the asymmetric unit in TPB-Ph is displayed. Hydrogen atoms are omitted for clarity.

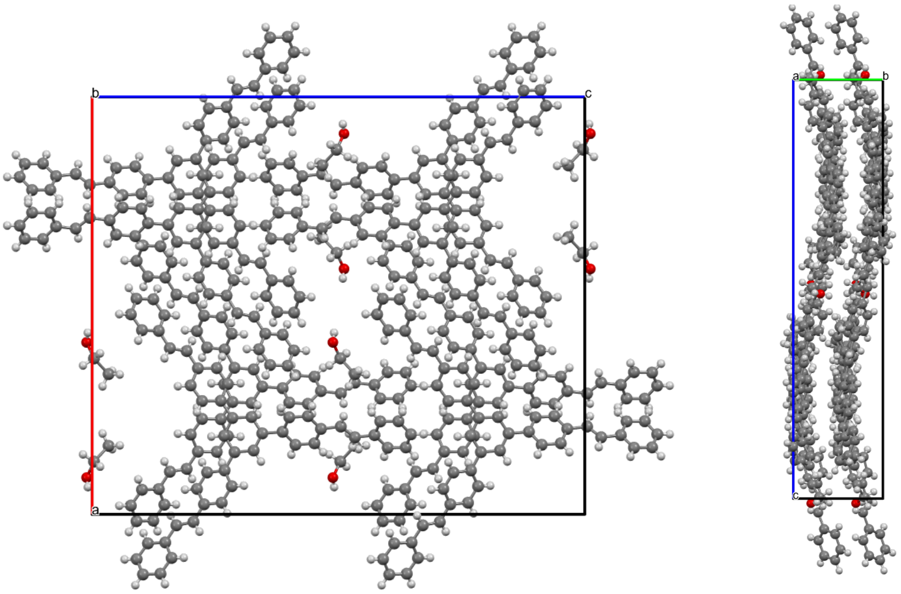

**Figure S24.** Single crystal X-ray structure of TPB-Ph viewed along c-direction (left) and viewed along b-direction (right).

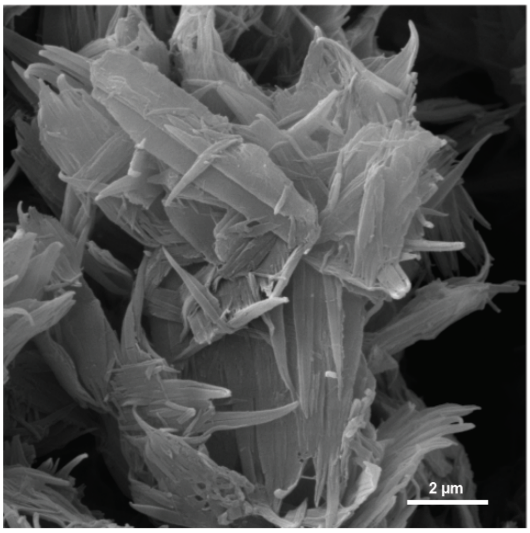


**Figure S25.** SEM image of TPB-Ph. Small crystallite powder sample was prepared, and its morphology was examined.

**S3.8. Synthesis and characterization of HKU-50-imine**

**Synthetic method for HKU-50-imine:** A 10 mL pyrex tube was added with 1,3,5-tris(4-formylphenyl)benzene (TFPB, 23.4 mg, 0.03 mmol) and 1,3,5-tris(4-aminophenyl)benzene (TAPB, 21.1 mg, 0.03 mmol), dioxane (0.75 mL), mesitylene (0.25 mL), and AcOH (0.1 mL, 6 M), the mixture was sonicated until yellow homogenous suspension was obtained, degassed through three freeze–pump–thaw cycles. The system was heated at 120 °C for 7d. The resulting HKU-X imine crystals were washed with THF (20 mL, 3 times per day for 3 days), followed by solvent exchange with anhydrous EtOH (20 mL, 3 times per day for 3 days). The EtOH-exchanged HKU-X imine crystals were transferred into a Kimwipe bag and activated via supercritical $CO_2$ to yield yellow crystals. Similar structures were reported before with different models.[7,8] In the reported results, HKU-50-imine showed much lower stability under aqueous acidic conditions and no emission under solid state. [7]

**Table S3.** The fractional atomic coordinates and the unit cell parameters of HKU-50-imine, simulated based on PXRD of HKU-50-imine.

| **HKU-50-imine** | **Space group:** *P*3 | | | | |
|---|---|---|---|---|---|
| | $a = b = 25.2100$ Å, $c = 3.8100$ Å, $\alpha = \beta = 90°$, $\gamma = 120°$ | | | | |
| Atom site label | Atom site symbol | x | y | z | Atom site occupancy |
| C1 | C | 0.24010 | 0.80778 | 0.15304 | 1.00 |
| C2 | C | 0.27366 | 0.77950 | 0.06568 | 1.00 |
| C3 | C | 0.25510 | 0.71988 | 0.18460 | 1.00 |
| C4 | C | 0.19990 | 0.68822 | 0.37472 | 1.00 |
| C5 | C | 0.16552 | 0.71604 | 0.45329 | 1.00 |
| C6 | C | 0.18654 | 0.77688 | 0.35130 | 1.00 |
| C7 | C | 0.29499 | 0.69256 | 0.13356 | 1.00 |
| N8 | N | 0.65296 | 0.84225 | 0.46029 | 1.00 |
| C9 | C | 0.10316 | 0.22599 | 0.65250 | 1.00 |
| C10 | C | 0.06306 | 0.16486 | 0.73108 | 1.00 |
| C11 | C | 0.08287 | 0.13041 | 0.92467 | 1.00 |
| C12 | C | 0.14426 | 0.15944 | 1.03887 | 1.00 |
| C13 | C | 0.18489 | 0.22047 | 0.95196 | 1.00 |

| | | | | | |
|---|---|---|---|---|---|
| C14 | C | 0.16468 | 0.25401 | 0.75671 | 1.00 |
| C15 | C | 0.04052 | 0.06386 | 0.98530 | 1.00 |
| C16 | C | 0.97690 | 0.04000 | 0.99285 | 1.00 |
| C17 | C | 0.10992 | 0.79217 | 0.65903 | 1.00 |
| C18 | C | 0.26987 | 0.62880 | 0.12792 | 1.00 |
| H19 | H | 0.25624 | 0.85442 | 0.06860 | 1.00 |
| H20 | H | 0.31421 | 0.80470 | 0.09411 | 1.00 |
| H21 | H | 0.18446 | 0.64311 | 0.48167 | 1.00 |
| H22 | H | 0.12446 | 0.69043 | 0.60734 | 1.00 |
| H23 | H | 0.08625 | 0.25062 | 0.49936 | 1.00 |
| H24 | H | 0.01728 | 0.14439 | 0.62425 | 1.00 |
| H25 | H | 0.16108 | 0.13547 | 1.19822 | 1.00 |
| H26 | H | 0.23202 | 0.24160 | 1.03920 | 1.00 |
| H27 | H | 0.95907 | 0.07098 | 0.98637 | 1.00 |
| H28 | H | 0.08540 | 0.74603 | 0.76084 | 1.00 |
| H29 | H | 0.22078 | 0.59964 | 0.13481 | 1.00 |

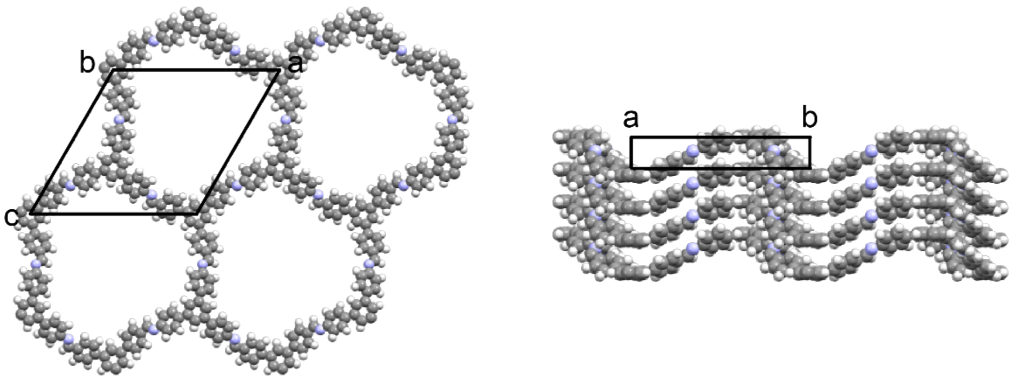


**Figure S26.** [001] (left), and [100] (right) views of HKU-50-imine structure.

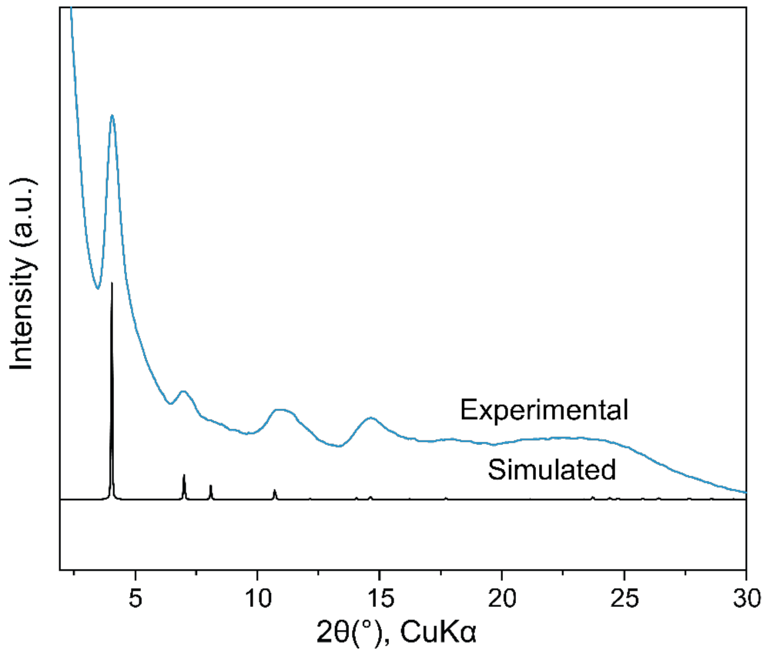


**Figure S27.** PXRD pattern of HKU-50-imine.

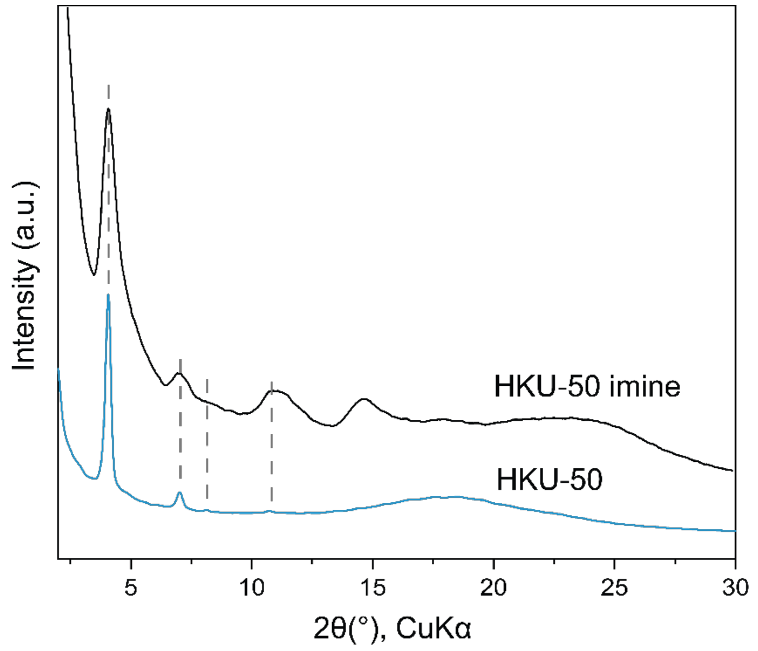


**Figure S28.** PXRD pattern comparison of HKU-50-imine and HKU-50.

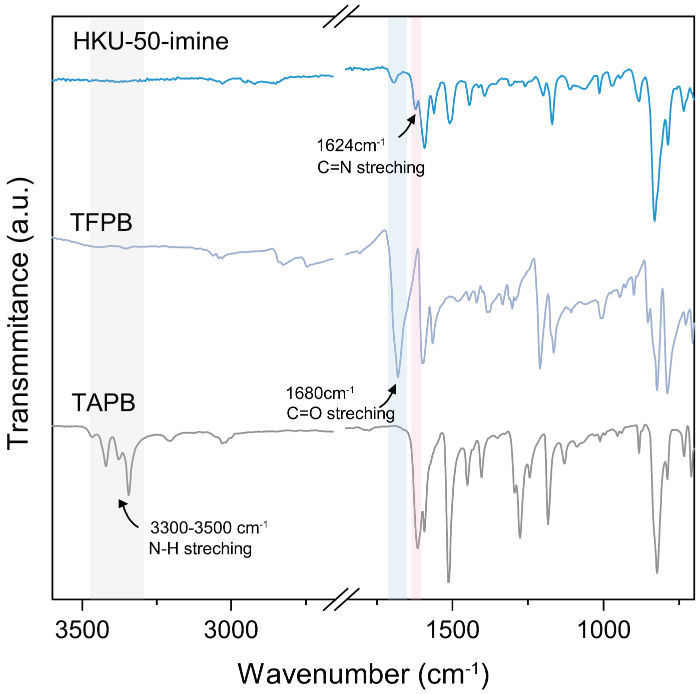


**Figure S29.** FT-IR of HKU-50-imine, TFPB, and TAPB.

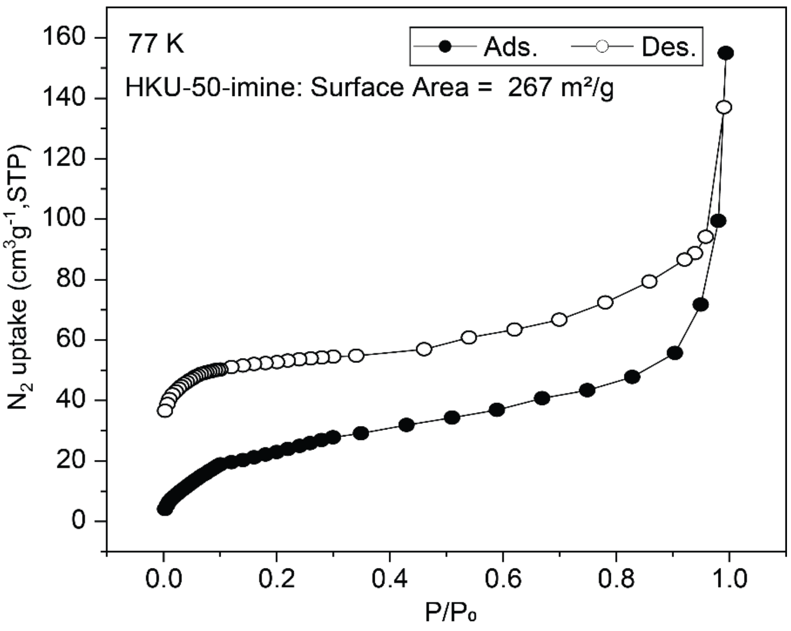


**Figure S30.** $N_2$ adsorption isotherms on HKU-50-imine measured at 77 K, surface area: 267 $m^2/g$.

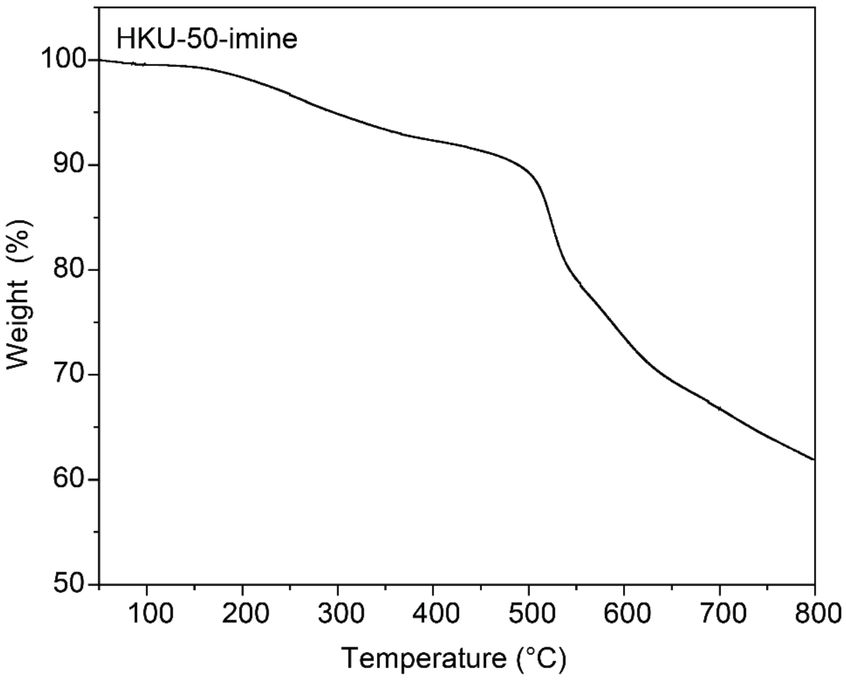


**Figure S31.** Thermal gravimetric analysis of HKU-50-imine.

## S3.9. ICP-OES measurement

**Digestion method for ICP-OES:** Samples ranging in mass from 10 to 15 mg were digested in Parr instrument A280AC vessels placed in a heat oven (Fisherbrand™ 180L Gravity Oven) with 6 mL of 65% $HNO_3$ (Suprapur™, MilliporeSigma™). Samples were pre-digested before heat by aging the sample mixed with acids for 16 h at room temperature. Then, the oven system was set at 130 °C for 10 hours. Final digests were diluted with deionized water to 10 ml stock solution in volumetric flasks and measured within 24 h.

**ICP-OES measurement**: Calibration curve was obtained by measuring a series of standard solutions with concentrations ranging from 0.001 mg/L to 5.000 mg/L (prepared by diluting a standard solution containing 10 mg/L of ruthenium in a matrix of 10% HCl and 1% $HNO_3$, PerkinElmer TruQms). Measure a 3% nitric acid solution two times as the measurement blanks. The apparatus was purged with 3% $HNO_3$ for 90 s after each sample and the signal from each sample was allowed to stabilize for 30 s before each measurement. Three measurements were obtained for each sample and averaged. The limit of quantification calculated from obtained results for blank solutions was 0.03 ppm. HKU-50: Ru weight percentage in sample: 0.0235% on average. The adsorption capacity of Ru was calculated by the following equation (1):

$$\textit{Weight percentage}\ (\%) = \frac{C_0(mg/L)*f*V_0(mL)*10^{-9}}{m(g)*10^{-3}}*100\%$$

$$\textit{Ru}\ (240.373\ \text{nm, ppm}) = \frac{C_0(mg/L)*f*V_0(mL)*10^{-3}}{m(g)*10^{-3}}$$

where $C_0$ (mg/L) is the initial concentration of Ru, $V_0$ (mL) is the volume of the solution, m (g) is the weight of the sample.

**Table S4.** Summary of ICP data

| Sample | Ru (240.272 nm, ppm) | Weight percentage (%) |
|---|---|---|
| HKU-50 | 224±3 | 0.0224±0.0003 |

## S4. Crystallization study with stilbene

### S4.1. Studies of the metathesis process in a molecular system

**Secondary metathesis of internal stilbene molecules:** One NMR tube was charged with *cis*-stilbene (0.1 mmol) and G2 (4 μmol, 3.4 mg), followed by vacuum and Argon refilling three times. Under Ar atmosphere, $CDCl_3$ (0.5 ml) was added to the tube, followed by $^1H$ NMR measurement immediately. Then, the tube was placed in the oil bath at 35 °C and allowed to react undisturbedly for 4 hours, after which time the $^1H$ NMR was also measured.

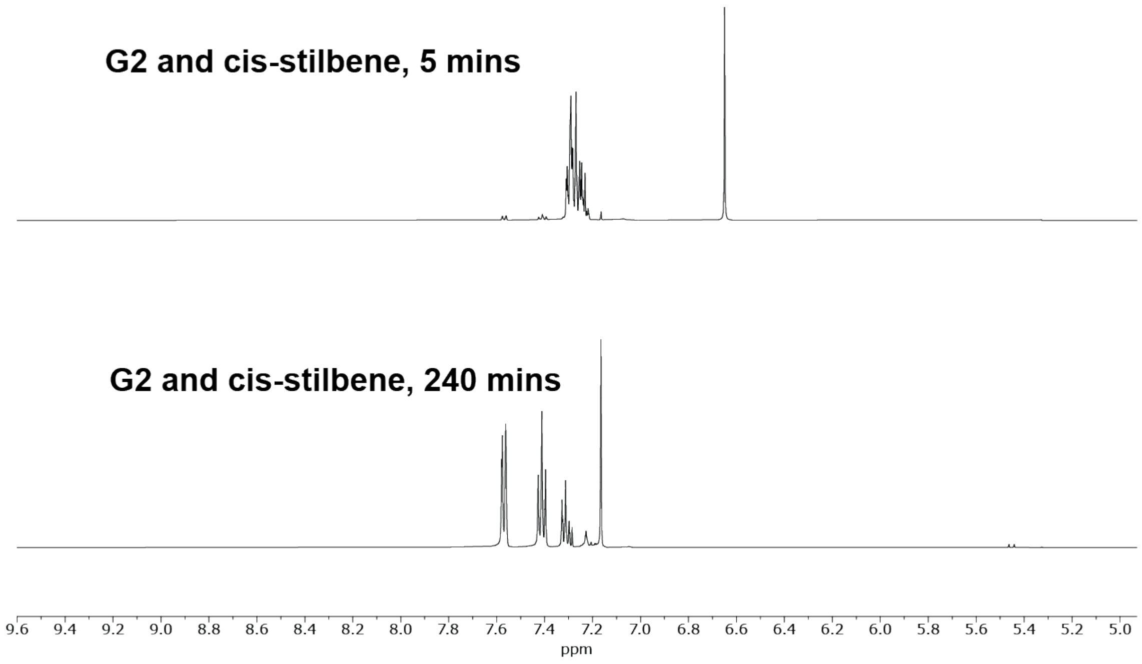


**Figure S32.** $^1H$ NMR Spectrum (400 MHz, $CDCl_3$, 298 K) of model test using *cis*-stilbene and G2 under homogenous state.

**NMR studies of the existence of Ru active species during the error-correcting process:** Two NMR tubes were charged with TPB-Me (8 μmol, 3.5mg) and G2 (4 μmol, 3.4 mg), and one of them was added with trans-stilbene (7.5 μmol, 1.4 mg). Both tubes went through vacuum and Argon refilling three times. Under Ar atmosphere, $CDCl_3$ (0.5 ml) was added to the tube, followed by $^1H$ NMR and $^{31}P$ NMR measurement immediately. Then, the tube was placed in the water bath at 30 °C and allowed to react undisturbedly. Measure the $^1H$ NMR and $^{31}P$ NMR of both reaction mixtures at various desired time intervals.

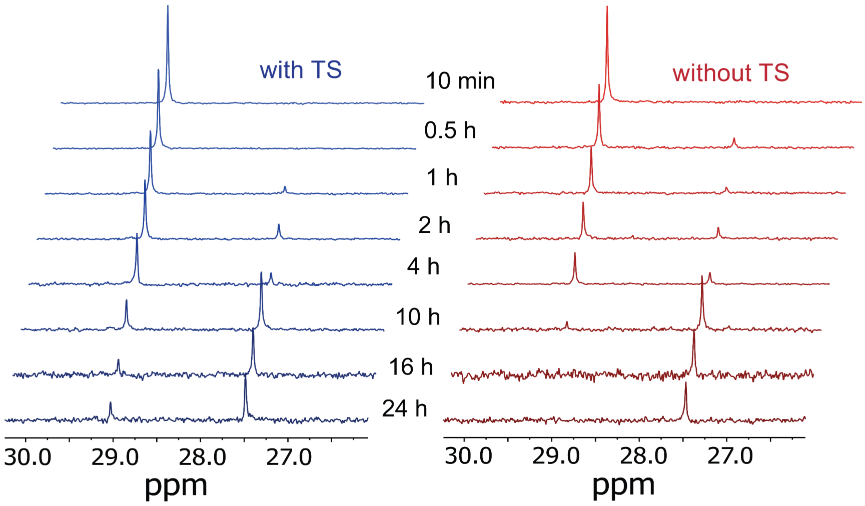


**Figure S33.** $^{31}P$ NMR Spectrum (162 MHz, $CDCl_3$, 298 K) of model test using (left) TPB-Me, TS, and G2, (right) TPB-Me, and G2 under room temperature.

**NMR studies of reaction of Ru species in amorphous with TS during the error-correcting process:** One Schlenk tube was charged with TPB-Me (0.05 mmol), and G2 (4 μmol, 3.4 mg) in $CDCl_3$ (0.5 ml). Both tubes went through immediate degassing via freeze-pump-thaw three times and Argon refilling. Then the vial was put under Ar atmosphere at 60 °C for 5 days. After cooling down, degassed $CDCl_3$ (0.4 ml) was added to the tube. 0.2 ml supernatant was first sampled for $^1H$ NMR measurement immediately. Then, TS (100 μmol) was added to the tube and placed in a water bath at 60 °C again, where it was allowed to react undisturbed for 1 hour. Measure the $^1H$ NMR of the supernatant again in the tube after cooling down.

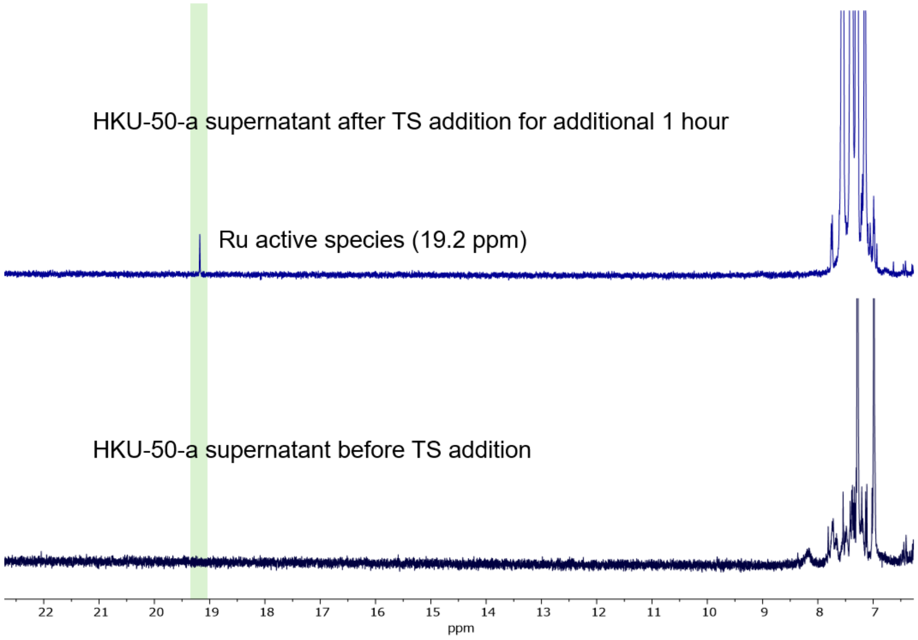


**Figure S34.** $^1H$ NMR of HKU-50-a supernatant before TS addition in the tube (bottom) and HKU-50-a supernatant in the tube after addition of TS with one-hour reaction (top).

**S4.2. PXRD study of the influence of the amount of TS on the crystallinity of HKU-50.**

**Set-up of multiple additions of modulator in synthesis of HKU-50:** Three 10 ml Schlenk tubes were charged with building units (0.05 mmol), and G2 (4 μmol, 3.4 mg). DCE (0.5 ml for TPB-Me), followed by immediately degassing via freeze-pump-thaw three times before refilling the tube with 1 atm Argon. Upon warming up to room temperature, the tubes were placed in the oil bath at 60 °C and allowed to react undisturbedly for 7 days. Then, one tube was diluted with a mixture of DMSO/THF (50 ml, v/v=1/10) and further washed with THF (10 ml, 3 times per day for 3 days). The THF-washed crystals were solvent-exchanged with anhydrous MeOH (10 ml, 3 times per day for 3 days) for PXRD. In the other 2 tubes, one of them was added with G2 (1 mg), and the other one was charged with G2 (1mg) and TS (3.2 mg), followed by freeze-pump-thaw three times before refilling the tube with 1 atm Argon. These two tubes were placed in the oil bath at 60 °C and allowed to react undisturbedly for 7 more days. Then the tubes were quenched with mixture of DMSO/THF (50 ml, v/v=1/10) and further washed with THF (10 ml, 3 times per day for 3 days). The THF-washed crystals were solvent-exchanged with anhydrous MeOH (10 ml, 3 times per day for 3 days) for PXRD.

## S5. Photoluminescence studies.

### S5.1. Photoluminescence measurement of HKU-50, HKU-50-a, TPB-Me, and TPB-Ph

**Femtosecond Fluorescence Up-conversion Spectrometer**

Femtosecond time-resolved fluorescence up-conversion (fs-FU) spectra of COFs was performed by regenerative amplifier Ti:Sapphire laser system (Spitfire-Pro, Spectra-Physics Company) and automated data acquisition and analysis spectrometer (Ultrafast, Helios).[9] The amplifier produced a 120 fs pulse laser at 800 nm (repetition rate at 1 kHz, average output energy was 6.2 W), which was divided into two beams: one beam, through the Optical Parametric Amplifiers (OPA), became the pump pulse (365 nm) which excited the fluorescence of the sample on the cuvette stage. Another beam, as the probe pulse, went through delay stage and was focused into the nonlinear crystal β-$BaB_2O_4$ (BBO) where it overlapped with the fluorescence to generate the sum frequency (SF) signal. Finally, by switching the BBO crystal angles, SF signals were collected by the fiber coupling to multi-channel photomultiplier tube (PMT) and analyzed by the computer. The instrument response function (IRF) for the fs-FU is 120 fs.

The fluorescence decay kinetics of these samples are proceeded with biexponential fitting by the following equation:

$$S(t)=\frac{1}{\tau_p\sqrt{\pi}}e^{-(\frac{\tau-\tau_0}{\tau_p})^2} * \sum_i A_i e^{-\frac{\tau-\tau_0}{\tau_i}} = \frac{1}{\tau_p\sqrt{\pi}}e^{-(\frac{\tau-\tau_0}{\tau_p})^2} * (A_1 e^{-\frac{\tau-\tau_0}{\tau_1}} + A_2 e^{-\frac{\tau-\tau_0}{\tau_2}}), \tau_p = \frac{IRF}{2\sqrt{ln2}}$$

where $\tau_1$ and $\tau_2$ are the fluorescent lifetimes, $A_1$ and $A_2$ are the corresponding amplitudes. The average fluorescent lifetimes are calculated by following equation:

$$\tau=\frac{A_1\tau_1^2+A_2\tau_2^2}{A_1\tau_1+A_2\tau_2}$$

**Steady-state fluorescence spectroscopy**

The steady-state fluorescence spectra and the photoluminescence (PL) quantum yield (PLQY) were measured by FLS 1000 PL Spectrometer (Edinburgh, UK), where the powder was tested by two quartz clips with a thickness of 2 mm, one of which has a 1 mm thick groove.

**Ultraviolet–visible diffuse reflectance spectroscopy (UV–vis DRS)**

UV-vis diffuse reflectance spectroscopy (DRS) was recorded by Cary 5000 UV-Vis-NIR spectrometer (Agilent, USA). The sample holder is a 2mm thick quartz with an elastic metal pressure block, with $BaSO_4$ as background.

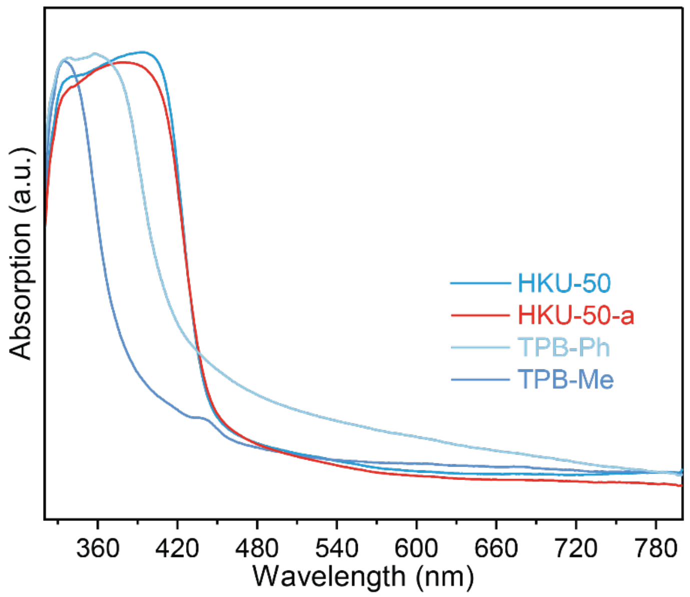


**Figure S35.** Ultraviolet–visible diffuse reflectance spectroscopy (UV–vis DRS) of HKU-50, HKU-50-a, TPB-Me, and TPB-Ph.

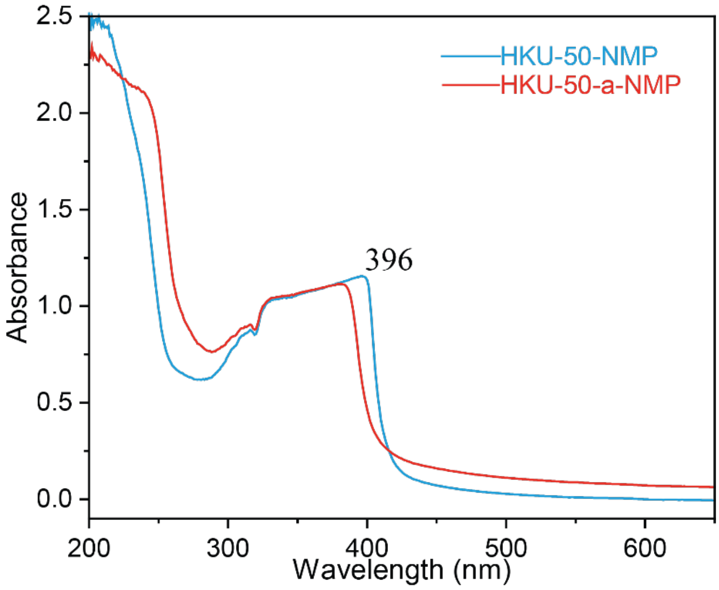


**Figure S36.** UV–vis DRS of HKU-50 and HKU-50-a in NMP.

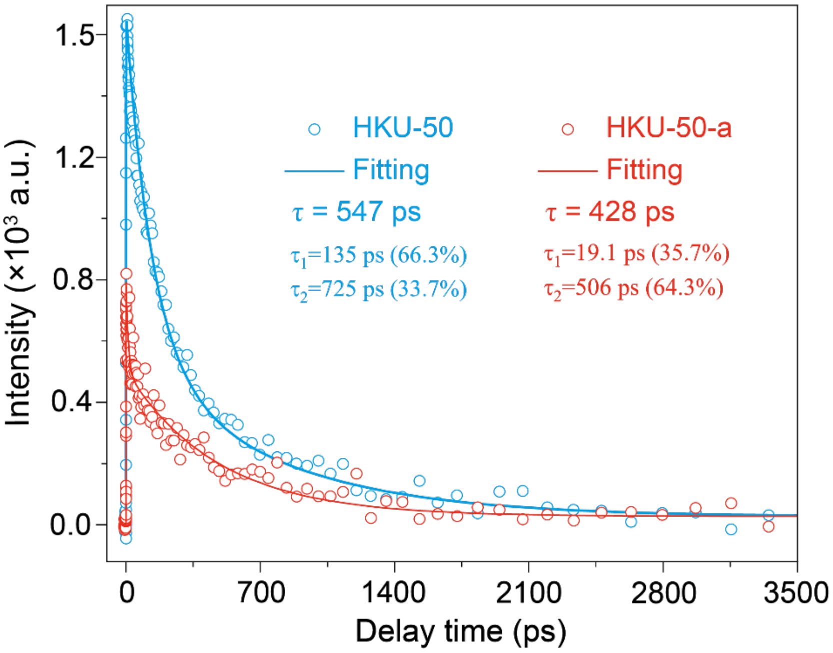


**Figure S37.** The fluorescence decay kinetics and fitting plots of HKU-50 and HKU-50-a with $\tau_1$ and $\tau_2$.

**S5.2. Confocal fluorescence images of HKU-50 and HKU-50-a.**

**Confocal fluorescence microscopy:** All fluorescence measurements were carried out at room temperature on a Confocal Imaging Sys, Leica TCS SP8. The testing samples were excited at 405 nm, and the emission spectrum was scanned from 430 to 480 nm. The solid samples of COFs were packed onto a square quartz plate with an approximate thickness of 1 mm.

# S6. Density functional theory (DFT) computational study

## S6.1. Electronic structure analysis

Density functional theory (DFT) calculations were performed using the Gaussian 16 program package to investigate the electronic structure and vibrational properties of the TPB-Me building unit and its extended oligomers.[10] The molecular geometries were directly obtained from the X-ray crystallographic data without further optimization to preserve the experimentally observed structural features. Single-point energy calculations and harmonic frequency analyses were carried out at the B3LYP/6-31+G(d) level of theory for the monomer, dimer, and trimer structures. The SCF convergence was achieved using the XQC algorithm with a maximum of 300 cycles to ensure reliable convergence for these extended conjugated systems. The calculated vibrational frequencies were scaled by a factor of 0.97 to account for anharmonicity and basis set limitations. The HOMO-LUMO gaps were calculated for the monomer, dimer, and trimer to evaluate the effect of conjugation length on the electronic properties. The monomer exhibits a HOMO-LUMO gap of 0.1698 Hartree (4.62 eV), which decreases to 0.1317 Hartree (3.58 eV) for the dimer and further to 0.1294 Hartree (3.52 eV) for the trimer. This progressive reduction in the bandgap with increasing conjugation length is consistent with the red shift observed in the UV-Vis diffuse reflectance spectra when comparing TPB-Me to HKU-50.

Analysis of the molecular orbital distributions reveals that the HOMO of the monomer is primarily localized on the central triphenylbenzene core and the vinylene moieties. Upon extension to the dimer and trimer, the HOMO becomes progressively delocalized across the entire molecular backbone through the vinylene linkages. This extended π-conjugation provides a theoretical basis for the enhanced optical properties observed in the crystalline COF.

## S6.2. Vibrational analysis

The calculated IR spectra show several notable trends that support the successful formation of the extended framework. The C=C stretching vibration in the 1500-1600 $cm^{-1}$ region exhibits a significant increase in intensity from the monomer to the trimer, reflecting the growing contribution of vinylene linkages to the vibrational modes as the conjugation extends. Additionally, the relative intensity of the C-H stretching bands near 3000 $cm^{-1}$ decreases progressively from the monomer to the trimer, which is attributed to the decreasing proportion of terminal methyl groups in the extended structures. This computational observation is in good agreement with the experimental FT-IR data, where the

characteristic methyl C-H stretching band at approximately 2900 $cm^{-1}$ present in TPB-Me is absent in HKU-50.

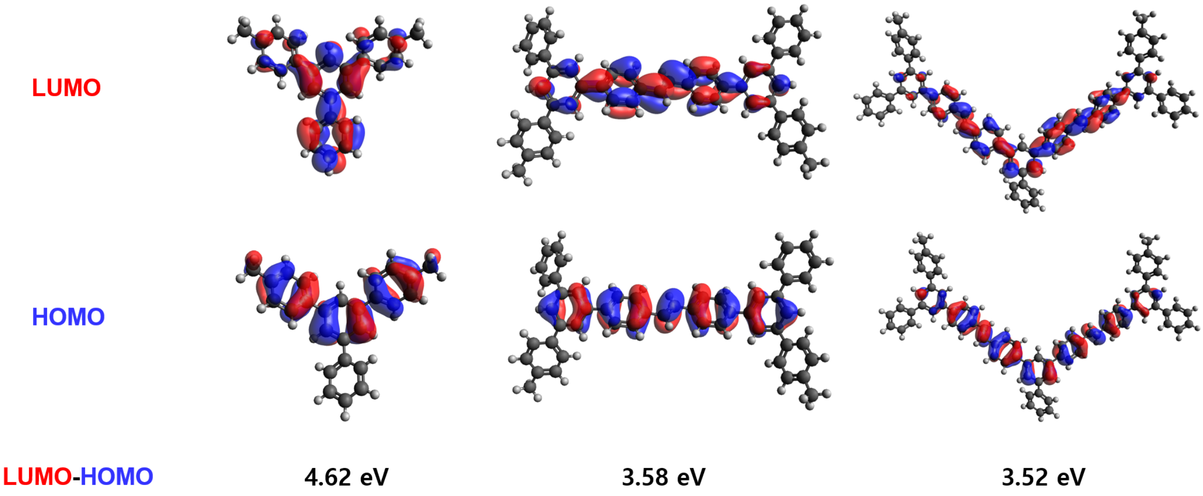


**Figure S38.** Highest occupied molecular orbitals (HOMOs) and lowest unoccupied molecular orbitals (LUMOs) of the monomer, dimer, and trimer via DFT calculation.

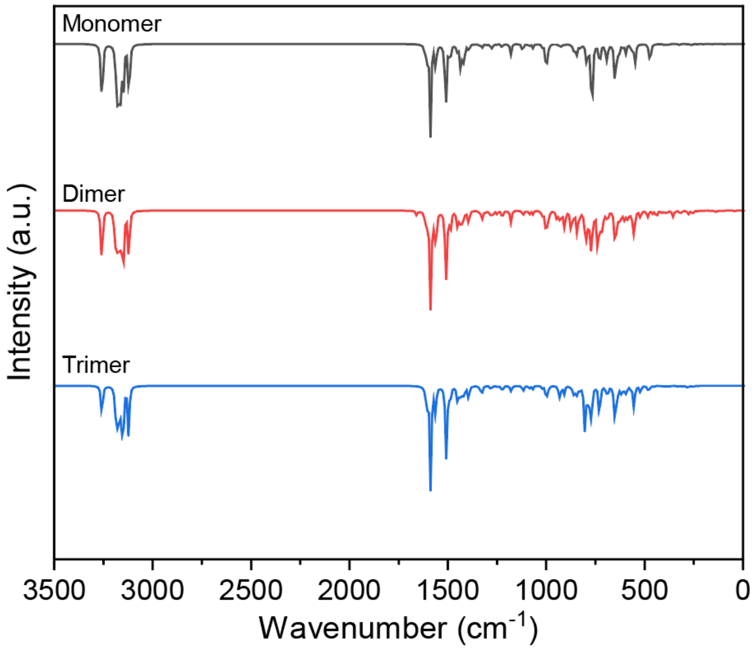


**Figure S39.** FT-IR of monomer (top), dimer (middle), and trimer (bottom) via DFT calculation.

**S6.3. Coordinates of studied molecules**

Monomer

| | | | |
|---|---|---|---|
| C | 9.49065550 | 32.47029169 | 1.28726922 |
| C | 8.29465550 | 33.20129169 | 1.32626922 |
| C | 9.42665550 | 35.45329169 | 1.27926922 |
| H | 10.22365550 | 35.06929169 | 0.65726922 |

| | | | |
|---|---|---|---|
| H | 10.40665550 | 37.34329169 | 1.47426922 |
| H | 10.43265550 | 32.99229169 | 1.31526922 |
| C | 7.07865550 | 32.50429169 | 1.28726922 |
| C | 8.25565550 | 30.39829169 | 1.28726922 |
| C | 7.04365550 | 31.10329169 | 1.32626922 |
| C | 9.48665550 | 31.06829169 | 1.32626922 |
| C | 4.52665550 | 30.95729169 | 1.27926922 |
| C | 10.87165550 | 28.96129169 | 1.27926922 |
| H | 4.46165550 | 31.83929169 | 0.65726922 |
| H | 10.14065550 | 28.46429169 | 0.65726922 |
| H | 2.40065550 | 30.86029169 | 1.47426922 |
| H | 12.01765550 | 27.16829169 | 1.47426922 |
| H | 6.15565550 | 33.05829169 | 1.31526922 |
| H | 8.23665550 | 29.32129169 | 1.31526922 |
| H | 1.34619211 | 29.47509663 | 3.02635084 |
| C | 8.32065550 | 34.65829169 | 1.62826922 |
| C | 9.52565550 | 36.76729169 | 1.73226922 |
| C | 8.50365550 | 37.33729169 | 2.50426922 |
| C | 7.35765550 | 36.57229169 | 2.78326922 |
| C | 7.28265550 | 35.24629169 | 2.37726922 |
| H | 6.52565550 | 36.95929169 | 3.34926922 |
| H | 6.42965550 | 34.67629169 | 2.71626922 |
| C | 5.76865550 | 30.39629169 | 1.62826922 |
| C | 10.73565550 | 30.31729169 | 1.62826922 |
| C | 3.33965550 | 30.38529169 | 1.73226922 |
| C | 11.95965550 | 28.21929169 | 1.73226922 |
| C | 3.35765550 | 29.21529169 | 2.50426922 |
| C | 12.96465550 | 28.82029169 | 2.50426922 |
| C | 4.59265550 | 28.60529169 | 2.78326922 |
| C | 12.87465550 | 30.19429169 | 2.78326922 |
| C | 5.77865550 | 29.20329169 | 2.37726922 |
| C | 11.76365550 | 30.92229169 | 2.37726922 |
| C | 2.06865550 | 28.68629169 | 2.99926922 |
| C | 14.06665550 | 27.96829169 | 2.99926922 |
| H | 4.67265550 | 27.69129169 | 3.34926922 |
| H | 13.62565550 | 30.72129169 | 3.34926922 |
| H | 6.69865550 | 28.74929169 | 2.71626922 |
| H | 11.69665550 | 31.94629169 | 2.71626922 |
| H | 14.32093633 | 27.24480907 | 2.25306941 |
| H | 8.59426697 | 38.33704812 | 2.87464286 |
| H | 14.92058335 | 28.57716293 | 3.21136128 |
| H | 13.75608615 | 27.46621641 | 3.89166324 |
| H | 2.20058162 | 28.28949586 | 3.98417966 |
| H | 1.72642576 | 27.91147905 | 2.34547706 |

Dimer

| | | | |
|---|---|---|---|
| C | 9.49065550 | 32.47029169 | 1.28726922 |

| | | | |
|---|---|---|---|
| C | 8.29465550 | 33.20129169 | 1.32626922 |
| C | 9.42665550 | 35.45329169 | 1.27926922 |
| H | 10.22365550 | 35.06929169 | 0.65726922 |
| H | 10.40665550 | 37.34329169 | 1.47426922 |
| H | 10.43265550 | 32.99229169 | 1.31526922 |
| C | 7.07865550 | 32.50429169 | 1.28726922 |
| C | 8.25565550 | 30.39829169 | 1.28726922 |
| C | 7.04365550 | 31.10329169 | 1.32626922 |
| C | 9.48665550 | 31.06829169 | 1.32626922 |
| C | 4.52665550 | 30.95729169 | 1.27926922 |
| C | 10.87165550 | 28.96129169 | 1.27926922 |
| H | 4.46165550 | 31.83929169 | 0.65726922 |
| H | 10.14065550 | 28.46429169 | 0.65726922 |
| H | 2.40065550 | 30.86029169 | 1.47426922 |
| H | 12.01765550 | 27.16829169 | 1.47426922 |
| H | 6.15565550 | 33.05829169 | 1.31526922 |
| H | 8.23665550 | 29.32129169 | 1.31526922 |
| H | -5.16714933 | 17.04943839 | 4.60328656 |
| C | -4.29734450 | 21.69029169 | 5.22726922 |
| C | -1.74634450 | 25.95229169 | 5.22726922 |
| C | -6.71334450 | 26.03129169 | 5.22726922 |
| C | -5.50334450 | 19.58129169 | 5.12426922 |
| C | 0.68265550 | 25.96329169 | 5.12426922 |
| C | -7.93734450 | 28.12929169 | 5.12426922 |
| C | -4.48034450 | 19.01129169 | 4.35226922 |
| C | 0.66465550 | 27.13329169 | 4.35226922 |
| C | -8.94134450 | 27.52929169 | 4.35226922 |
| C | -3.33534450 | 19.77629169 | 4.07326922 |
| C | -0.56934450 | 27.74329169 | 4.07326922 |
| C | -8.85234450 | 26.15429169 | 4.07326922 |
| C | -3.25934450 | 21.10229169 | 4.47926922 |
| C | -1.75634450 | 27.14529169 | 4.47926922 |
| C | -7.74134450 | 25.42629169 | 4.47926922 |
| C | -4.66734450 | 17.63129169 | 3.85726922 |
| C | 1.95365550 | 27.66229169 | 3.85726922 |
| H | -2.50334450 | 19.38929169 | 3.50726922 |
| H | -0.65034450 | 28.65729169 | 3.50726922 |
| H | -9.60334450 | 25.62829169 | 3.50726922 |
| H | -2.40734450 | 21.67229169 | 4.14026922 |
| H | -2.67534450 | 27.59929169 | 4.14026922 |
| H | -7.67434450 | 24.40329169 | 4.14026922 |
| H | 1.18765550 | 29.17929169 | 2.62226922 |
| C | 8.32065550 | 34.65829169 | 1.62826922 |
| C | 9.52565550 | 36.76729169 | 1.73226922 |
| C | 8.50365550 | 37.33729169 | 2.50426922 |
| C | 7.35765550 | 36.57229169 | 2.78326922 |

| | | | |
|---|---|---|---|
| C | 7.28265550 | 35.24629169 | 2.37726922 |
| H | 6.52565550 | 36.95929169 | 3.34926922 |
| H | 6.42965550 | 34.67629169 | 2.71626922 |
| C | 5.76865550 | 30.39629169 | 1.62826922 |
| C | 10.73565550 | 30.31729169 | 1.62826922 |
| C | 3.33965550 | 30.38529169 | 1.73226922 |
| C | 11.95965550 | 28.21929169 | 1.73226922 |
| C | 3.35765550 | 29.21529169 | 2.50426922 |
| C | 12.96465550 | 28.82029169 | 2.50426922 |
| C | 4.59265550 | 28.60529169 | 2.78326922 |
| C | 12.87465550 | 30.19429169 | 2.78326922 |
| C | 5.77865550 | 29.20329169 | 2.37726922 |
| C | 11.76365550 | 30.92229169 | 2.37726922 |
| C | 2.06865550 | 28.68629169 | 2.99926922 |
| C | 14.06665550 | 27.96829169 | 2.99926922 |
| H | 4.67265550 | 27.69129169 | 3.34926922 |
| H | 13.62565550 | 30.72129169 | 3.34926922 |
| H | 6.69865550 | 28.74929169 | 2.71626922 |
| H | 11.69665550 | 31.94629169 | 2.71626922 |
| H | 14.32093633 | 27.24480907 | 2.25306941 |
| H | 2.83565550 | 27.16929169 | 4.23326922 |
| C | -5.46834450 | 23.87829169 | 5.56926922 |
| C | -3.05634450 | 23.84529169 | 5.56926922 |
| C | -4.23334450 | 25.95129169 | 5.56926922 |
| C | -4.27234450 | 23.14829169 | 5.53026922 |
| C | -3.02134450 | 25.24629169 | 5.53026922 |
| C | -5.46334450 | 25.28029169 | 5.53026922 |
| C | -5.40434450 | 20.89529169 | 5.57626922 |
| C | -0.50434450 | 25.39229169 | 5.57626922 |
| C | -6.84834450 | 27.38729169 | 5.57626922 |
| H | -6.20134450 | 21.27929169 | 6.19826922 |
| H | -0.43834450 | 24.51029169 | 6.19826922 |
| H | -6.11734450 | 27.88529169 | 6.19826922 |
| H | -6.38434450 | 19.00629169 | 5.38226922 |
| H | 1.62165550 | 25.48829169 | 5.38226922 |
| H | -7.99534450 | 29.18029169 | 5.38226922 |
| H | -6.41034450 | 23.35629169 | 5.54126922 |
| H | -2.13234450 | 23.29029169 | 5.54126922 |
| H | -4.21434450 | 27.02729169 | 5.54126922 |
| H | 8.59426697 | 38.33704812 | 2.87464286 |
| H | -9.76158485 | 28.10779853 | 3.98154614 |
| H | -3.71312316 | 17.19597015 | 3.64547499 |
| H | -5.25714268 | 17.64939317 | 2.96468284 |
| H | 14.92058335 | 28.57716293 | 3.21136128 |
| H | 13.75608615 | 27.46621641 | 3.89166324 |

Trimer

| | | | |
|---|---|---|---|
| C | 9.49065550 | 32.47029169 | 1.28726922 |
| C | 8.29465550 | 33.20129169 | 1.32626922 |
| C | 9.42665550 | 35.45329169 | 1.27926922 |
| H | 10.22365550 | 35.06929169 | 0.65726922 |
| H | 10.40665550 | 37.34329169 | 1.47426922 |
| H | 10.43265550 | 32.99229169 | 1.31526922 |
| C | 7.07865550 | 32.50429169 | 1.28726922 |
| C | 8.25565550 | 30.39829169 | 1.28726922 |
| C | 7.04365550 | 31.10329169 | 1.32626922 |
| C | 9.48665550 | 31.06829169 | 1.32626922 |
| C | 4.52665550 | 30.95729169 | 1.27926922 |
| C | 10.87165550 | 28.96129169 | 1.27926922 |
| H | 4.46165550 | 31.83929169 | 0.65726922 |
| H | 10.14065550 | 28.46429169 | 0.65726922 |
| H | 2.40065550 | 30.86029169 | 1.47426922 |
| H | 12.01765550 | 27.16829169 | 1.47426922 |
| H | 6.15565550 | 33.05829169 | 1.31526922 |
| H | 8.23665550 | 29.32129169 | 1.31526922 |
| H | -5.16714933 | 17.04943839 | 4.60328656 |
| H | 19.88735545 | 17.04958388 | 4.60306804 |
| C | -4.29734450 | 21.69029169 | 5.22726922 |
| C | -1.74634450 | 25.95229169 | 5.22726922 |
| C | -6.71334450 | 26.03129169 | 5.22726922 |
| C | -5.50334450 | 19.58129169 | 5.12426922 |
| C | 0.68265550 | 25.96329169 | 5.12426922 |
| C | -7.93734450 | 28.12929169 | 5.12426922 |
| C | -4.48034450 | 19.01129169 | 4.35226922 |
| C | 0.66465550 | 27.13329169 | 4.35226922 |
| C | -8.94134450 | 27.52929169 | 4.35226922 |
| C | -3.33534450 | 19.77629169 | 4.07326922 |
| C | -0.56934450 | 27.74329169 | 4.07326922 |
| C | -8.85234450 | 26.15429169 | 4.07326922 |
| C | -3.25934450 | 21.10229169 | 4.47926922 |
| C | -1.75634450 | 27.14529169 | 4.47926922 |
| C | -7.74134450 | 25.42629169 | 4.47926922 |
| C | -4.66734450 | 17.63129169 | 3.85726922 |
| C | 1.95365550 | 27.66229169 | 3.85726922 |
| H | -2.50334450 | 19.38929169 | 3.50726922 |
| H | -0.65034450 | 28.65729169 | 3.50726922 |
| H | -9.60334450 | 25.62829169 | 3.50726922 |
| H | -2.40734450 | 21.67229169 | 4.14026922 |
| H | -2.67534450 | 27.59929169 | 4.14026922 |
| H | -7.67434450 | 24.40329169 | 4.14026922 |
| H | 1.18765550 | 29.17929169 | 2.62226922 |
| C | 8.32065550 | 34.65829169 | 1.62826922 |
| C | 9.52565550 | 36.76729169 | 1.73226922 |

| | | | |
|---|---|---|---|
| C | 8.50365550 | 37.33729169 | 2.50426922 |
| C | 7.35765550 | 36.57229169 | 2.78326922 |
| C | 7.28265550 | 35.24629169 | 2.37726922 |
| H | 6.52565550 | 36.95929169 | 3.34926922 |
| H | 6.42965550 | 34.67629169 | 2.71626922 |
| C | 5.76865550 | 30.39629169 | 1.62826922 |
| C | 10.73565550 | 30.31729169 | 1.62826922 |
| C | 20.75665550 | 21.69029169 | 5.22726922 |
| C | 23.30865550 | 25.95229169 | 5.22726922 |
| C | 18.34165550 | 26.03129169 | 5.22726922 |
| C | 3.33965550 | 30.38529169 | 1.73226922 |
| C | 11.95965550 | 28.21929169 | 1.73226922 |
| C | 19.55165550 | 19.58129169 | 5.12426922 |
| C | 25.73765550 | 25.96329169 | 5.12426922 |
| C | 17.11765550 | 28.12929169 | 5.12426922 |
| C | 3.35765550 | 29.21529169 | 2.50426922 |
| C | 12.96465550 | 28.82029169 | 2.50426922 |
| C | 20.57465550 | 19.01129169 | 4.35226922 |
| C | 25.71965550 | 27.13329169 | 4.35226922 |
| C | 16.11365550 | 27.52929169 | 4.35226922 |
| C | 4.59265550 | 28.60529169 | 2.78326922 |
| C | 12.87465550 | 30.19429169 | 2.78326922 |
| C | 21.71965550 | 19.77629169 | 4.07326922 |
| C | 24.48465550 | 27.74329169 | 4.07326922 |
| C | 16.20265550 | 26.15429169 | 4.07326922 |
| C | 5.77865550 | 29.20329169 | 2.37726922 |
| C | 11.76365550 | 30.92229169 | 2.37726922 |
| C | 21.79465550 | 21.10229169 | 4.47926922 |
| C | 23.29865550 | 27.14529169 | 4.47926922 |
| C | 17.31365550 | 25.42629169 | 4.47926922 |
| C | 2.06865550 | 28.68629169 | 2.99926922 |
| C | 14.06665550 | 27.96829169 | 2.99926922 |
| C | 20.38765550 | 17.63129169 | 3.85726922 |
| C | 15.01065550 | 28.38029169 | 3.85726922 |
| H | 4.67265550 | 27.69129169 | 3.34926922 |
| H | 13.62565550 | 30.72129169 | 3.34926922 |
| H | 22.55165550 | 19.38929169 | 3.50726922 |
| H | 24.40465550 | 28.65729169 | 3.50726922 |
| H | 15.45165550 | 25.62829169 | 3.50726922 |
| H | 6.69865550 | 28.74929169 | 2.71626922 |
| H | 11.69665550 | 31.94629169 | 2.71626922 |
| H | 22.64765550 | 21.67229169 | 4.14026922 |
| H | 22.37865550 | 27.59929169 | 4.14026922 |
| H | 17.38065550 | 24.40329169 | 4.14026922 |
| H | 14.08065550 | 26.95829169 | 2.62226922 |
| H | 2.83565550 | 27.16929169 | 4.23326922 |

| | | | |
|---|---|---|---|
| H | 14.99665550 | 29.39029169 | 4.23326922 |
| C | -5.46834450 | 23.87829169 | 5.56926922 |
| C | -3.05634450 | 23.84529169 | 5.56926922 |
| C | -4.23334450 | 25.95129169 | 5.56926922 |
| C | -4.27234450 | 23.14829169 | 5.53026922 |
| C | -3.02134450 | 25.24629169 | 5.53026922 |
| C | -5.46334450 | 25.28029169 | 5.53026922 |
| C | -5.40434450 | 20.89529169 | 5.57626922 |
| C | -0.50434450 | 25.39229169 | 5.57626922 |
| C | -6.84834450 | 27.38729169 | 5.57626922 |
| H | -6.20134450 | 21.27929169 | 6.19826922 |
| H | -0.43834450 | 24.51029169 | 6.19826922 |
| H | -6.11734450 | 27.88529169 | 6.19826922 |
| H | -6.38434450 | 19.00629169 | 5.38226922 |
| H | 1.62165550 | 25.48829169 | 5.38226922 |
| H | -7.99534450 | 29.18029169 | 5.38226922 |
| H | -6.41034450 | 23.35629169 | 5.54126922 |
| H | -2.13234450 | 23.29029169 | 5.54126922 |
| H | -4.21434450 | 27.02729169 | 5.54126922 |
| C | 19.58665550 | 23.87829169 | 5.56926922 |
| C | 21.99865550 | 23.84529169 | 5.56926922 |
| C | 20.82165550 | 25.95129169 | 5.56926922 |
| C | 20.78265550 | 23.14829169 | 5.53026922 |
| C | 22.03365550 | 25.24629169 | 5.53026922 |
| C | 19.59165550 | 25.28029169 | 5.53026922 |
| C | 19.65065550 | 20.89529169 | 5.57626922 |
| C | 24.55065550 | 25.39229169 | 5.57626922 |
| C | 18.20665550 | 27.38729169 | 5.57626922 |
| H | 18.85365550 | 21.27929169 | 6.19826922 |
| H | 24.61665550 | 24.51029169 | 6.19826922 |
| H | 18.93665550 | 27.88529169 | 6.19826922 |
| H | 18.67065550 | 19.00629169 | 5.38226922 |
| H | 26.67665550 | 25.48829169 | 5.38226922 |
| H | 17.05965550 | 29.18029169 | 5.38226922 |
| H | 18.64465550 | 23.35629169 | 5.54126922 |
| H | 22.92265550 | 23.29029169 | 5.54126922 |
| H | 20.84065550 | 27.02729169 | 5.54126922 |
| H | 26.63069352 | 27.55473594 | 3.98173652 |
| H | 8.59426697 | 38.33704812 | 2.87464286 |
| H | -9.76158485 | 28.10779853 | 3.98154614 |
| H | 21.34190555 | 17.19577772 | 3.64600063 |
| H | 19.79832381 | 17.64944012 | 2.96437572 |
| H | -3.71312316 | 17.19597015 | 3.64547499 |
| H | -5.25714268 | 17.64939317 | 2.96468284 |

**S7. References**

(1) Ma, J.-B.; Zhao, X.; Zhang, D.; Shi, S.-L. Enantio- and Regioselective Ni-Catalyzed para-C–H Alkylation of Pyridines with Styrenes via Intermolecular Hydroarylation. *J. Am. Chem. Soc.* **2022**, *144*, 13643-13651.
(2) Pastoetter, D. L.; Xu, S.; Borrelli, M.; Addicoat, M.; Biswal, B. P.; Paasch, S.; Dianat, A.; Thomas, H.; Berger, R.; Reineke, S.; Brunner, E.; Cuniberti, G.; Richter, M.; Feng, X. Synthesis of Vinylene-Linked Two-Dimensional Conjugated Polymers via the Horner–Wadsworth–Emmons Reaction. *Angew. Chem. Int. Ed.* **2020**, *59*, 23620-23625.
(3) Li, S.; Xu, S.; Lin, E.; Wang, T.; Yang, H.; Han, J.; Zhao, Y.; Xue, Q.; Samorì, P.; Zhang, Z.; Zhang, T. Synthesis of single-crystalline sp2-carbon-linked covalent organic frameworks through imine-to-olefin transformation. *Nat. Chem.* **2025**, *17*, 226-232.
(4) Sick, T.; Rotter, J. M.; Reuter, S.; Kandambeth, S.; Bach, N. N.; Döblinger, M.; Merz, J.; Clark, T.; Marder, T. B.; Bein, T.; et al. Switching on and off Interlayer Correlations and Porosity in 2D Covalent Organic Frameworks. *J. Am. Chem. Soc.* **2019**, *141*, 12570-12581.
(5) Sheldrick, G. Crystal structure refinement with shelxl. *Acta Crystallogr. C* **2015**, *71*, 3-8.
(6) Dolomanov, O. V.; Bourhis, L. J.; Gildea, R. J.; Howard, J. A. K.; Puschmann, H., OLEX2: a complete structure solution, refinement and analysis program. *J. Appl. Crystallogr.* **2009**, *42*, 339-341.
(7) Wei, D.; Zhao, W.; Xing, C.; Zhang, Y.; Li, H.; Zhi, Y. Emissive Covalent Organic Frameworks: Fluorescence Improvement via a Controllable Vertex Strategy and Chemical Sensing. *ACS Appl. Polym. Mater.* **2024**, *6*, 8498-8504.
(8) Wang, J.; Tao, Y.; Wang, D.; Wang, L.; Tian, M.; Yang, Y.; Liu, Q.; Zou, Y.; Zhou, Q.; Ke, F.; et al. Fabrication of highly crystalline covalent organic framework for solid-phase extraction of three dyes from food and water samples. *J. Sep. Sci.* **2023**, *46*, 2200996.
(9) Pang, J.; He, J.; Deng, Z.; Chen, W.; Chen, S.; Ni, S.; Phillips, D. L.; Dong, Z.; Dang, L.; Li, M.-D. Boosting Ultrafast Trans-Cis Photoisomerization and Intersystem Crossing in Nanocrystals of Double-Bond Photoswitching Molecules. *Adv. Optical Mater.* **2023**, *11*, 2300028.
(10) Frisch, M. J.; Trucks, G. W.; Schlegel, H. B.; Scuseria, G. E.; Robb, M. A.; Cheeseman, J. R.; Scalmani, G.; Barone, V.; Petersson, G. A.; Nakatsuji, H.; Li, X.; Caricato, M.; Marenich, A. V.; Bloino, J.; Janesko, B. G.; Gomperts, R.; Mennucci, B.; Hratchian, H. P.; Ortiz, J. V.; Izmaylov, A. F.; Sonnenberg, J. L.; Williams-Young, D.; Ding, F.; Lipparini, F.; Egidi, F.; Goings, J.; Peng, B.; Petrone, A.; Henderson, T.; Ranasinghe, D.; Zakrzewski, V. G.; Gao, J.; Rega, N.; Zheng, G.; Liang, W.; Hada, M.; Ehara, M.; Toyota, K.; Fukuda, R.; Hasegawa, J.; Ishida, M.; Nakajima, T.; Honda, Y.; Kitao, O.; Nakai, H.; Vreven, T.; Throssell, K.; Montgomery, J. A., Jr.; Peralta, J. E.; Ogliaro, F.; Bearpark, M. J.; Heyd, J. J.; Brothers, E. N.; Kudin, K. N.; Staroverov, V. N.; Keith, T. A.; Kobayashi, R.; Normand, J.; Raghavachari, K.; Rendell, A. P.; Burant, J. C.; Iyengar, S. S.; Tomasi, J.; Cossi, M.; Millam, J. M.; Klene, M.; Adamo, C.; Cammi, R.; Ochterski, J. W.; Martin, R. L.; Morokuma, K.; Farkas, O.; Foresman, J. B.; Fox, D. J. *Gaussian 09*, revision A.02; Gaussian, Inc.: Wallingford, CT, **2016**.
